# Thermoregulation of Meningococcal fHbp, an Important Virulence Factor and Vaccine Antigen, Is Mediated by Anti-ribosomal Binding Site Sequences in the Open Reading Frame

**DOI:** 10.1371/journal.ppat.1005794

**Published:** 2016-08-25

**Authors:** Edmund Loh, Hayley Lavender, Felicia Tan, Alexander Tracy, Christoph M. Tang

**Affiliations:** Sir William Dunn School of Pathology, University of Oxford, Oxford, United Kingdom; Institut Necker-Enfants-Malades, FRANCE

## Abstract

During colonisation of the upper respiratory tract, bacteria are exposed to gradients of temperatures. *Neisseria meningitidis* is often present in the nasopharynx of healthy individuals, yet can occasionally cause severe disseminated disease. The meningococcus can evade the human complement system using a range of strategies that include recruitment of the negative complement regulator, factor H (CFH) *via* factor H binding protein (fHbp). We have shown previously that fHbp levels are influenced by the ambient temperature, with more fHbp produced at higher temperatures (*i*.*e*. at 37°C compared with 30°C). Here we further characterise the mechanisms underlying thermoregulation of fHbp, which occurs gradually over a physiologically relevant range of temperatures. We show that fHbp thermoregulation is not dependent on the promoters governing transcription of the bi- or mono-cistronic *fHbp* mRNA, or on meningococcal specific transcription factors. Instead, fHbp thermoregulation requires sequences located in the translated region of the mono-cistronic *fHbp* mRNA. Site-directed mutagenesis demonstrated that two anti-ribosomal binding sequences within the coding region of the *fHbp* transcript are involved in fHbp thermoregulation. Our results shed further light on mechanisms underlying the control of the production of this important virulence factor and vaccine antigen.

## Author Summary

The bacterium *Neisseria meningitidis* is exquisitely adapted to survive in the human host, and possesses several mechanisms to interact with host cells in the upper airway and to circumvent immune responses. However, the mechanisms that govern the expression of factors that contribute to colonisation and disease are incompletely understood. In this work, we further characterise how temperature influences the production of factor H binding protein (fHbp) by the meningococcus; fHbp recruits human complement proteins to the surface of the bacterium, and is an important vaccine antigen. We show that thermoregulation of fHbp occurs gradually over a physiological range of temperatures found in the upper airway, the site of colonisation. This regulation does not require specific meningococcal transcription factors, and sequence analysis indicates that *fHbp* mRNA forms a secondary structure which could act as an RNA thermosensor. Additional studies demonstrate that there are two specific sequences within the coding region of *fHbp* mRNA are important for thermosensing and could base-pair to the ribosome binding site, thus blocking translation of this protein. As fHbp is thermoregulated, vaccines that target this antigen might not impose a high level of selective pressure on the bacterium at the mucosal surface, thereby limiting herd immunity induce by fHbp containing vaccines.

## Introduction

*Neisseria meningitidis* is a harmless member of the human nasopharyngeal flora in a significant proportion of healthy individuals [1]. However in some instances, the bacterium spreads from the upper airway into the systemic circulation, where it can replicate and spread to the rest of the body [2], especially the cerebrospinal fluid, resulting in meningitis. Therefore the meningococcus remains an important human pathogen in infants and young adults [3].

To survive in the human host, the meningococcus has evolved several mechanisms that enable it to evade the immune system [4]. In particular, the complement system is critical for protection against systemic *N*. *meningitidis* infection, evident from the increased susceptibility of individuals with defects in the complement system and findings from a genome wide association study [5,6]. The bacterium evades complement mediated killing by expressing a polysaccharide capsule, sialylation of its lipopolysaccharide, and by binding complement factor H (CFH), the major negative regulator of the alternative complement pathway [7]. CFH is recruited by high affinity interactions with factor H binding protein (fHbp) [8]. CFH competes with complement factor H related protein-3 (CFHR3) for binding to fHbp on the meningococcus [9]; CFHR3 is a competitive inhibitor of CFH binding to the meningococcal surface, and relative levels of CFH and CFHR3 in individuals are likely to determine host genetic susceptibility to meningococcal disease in the general population [9]. Furthermore fHbp is a surface lipoprotein which is an important component of two vaccines which are now licensed for preventing meningococcal disease [10,11].

Within the upper airway, the bacterium is exposed to gradients in ambient temperature that occur in relation to the anatomical location the phase in the respiratory cycle, and the presence of local inflammation [12,13]. The nasal epithelium contains a complex vascular network with a relatively high blood flow, which forms a heat exchanger, to condition inspired air; the temperature of air entering the nasopharynx rises rapidly from the nostrils, increasing to around 32–34°C by the time it reaches the glottis at the end of inspiration [14,15]. During colonisation, the meningococcus is found both on the epithelial surface as well as deep in the submucosal layer surface [16], so the bacterium will be will be exposed to a range of temperatures in these sites [17]. Additionally, during the development of invasive disease, *N*. *meningitidis* passes from the lower temperatures in the upper airway to the core body temperature of 37°C or higher with a febrile response to infection [18]. Therefore, temperature is likely to be an important environmental cue for the meningococcus during colonisation and the development of disease.

We have shown previously that three meningococcal genes, *cssA*, *fHbp*, and *lst* which encode factors contributing to capsule biosynthesis, fHbp, and LPS sialylation, respectively, are subject to thermoregulation. Of note, the sialic acid-capsule biosynthesis operon is controlled by an RNA thermosensor [19]. RNA thermosensors are usually located in the 5´-untranslated region (5´-UTR) of an mRNA, and form a secondary structure at lower temperatures that prevents protein translation by blocking access of ribosomes to the nascent mRNA. As the temperature rises, the secondary structure undergoes a conformational change, exposing the ribosome binding site (RBS), and allowing translation in response to the elevated temperature. RNA thermosensors have been described in an increasing number of microbes [20], especially enteric pathogens which are subject to a large fluctuation in temperature upon ingestion by mammalian hosts from the external environment.

Here we describe the mechanisms governing thermoregulation of fHbp. Expression of *fHbp* in *N*. *meningitidis* is initiated from two transcriptional start sites, resulting in a bi-cistronic transcript (including the upstream gene, *nmb1869*), or a mono-cistronic transcript from the *fHbp* promoter, P_*fHbp*_ [21]. It has been shown that transcription from P_*fHbp*_ is responsive to oxygen limitation, and regulated in an FNR-dependent manner [21]. We demonstrate that fHbp levels are governed by an RNA thermosensor and demonstrate that sequences in the coding region of *fHbp* contribute to thermosensing and translation efficiency; two sequences within the *fHbp* coding region, which are complementarity to the ribosome-binding site, are necessary for the thermoregulation of this key virulence factor and vaccine antigen.

## Results

### fHbp expression is temperature-dependent

**Fig 1:**
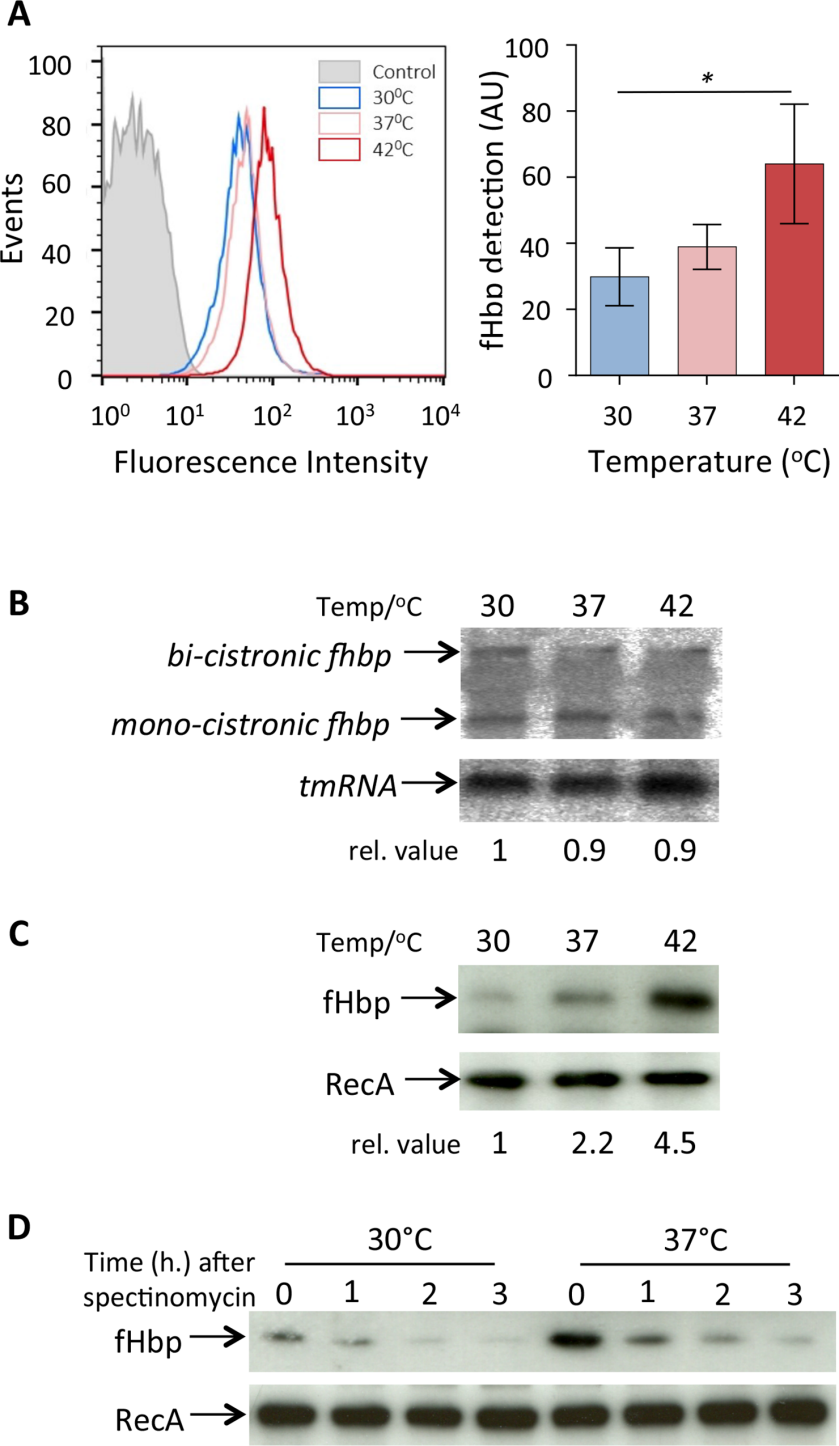
Thermoregulation of fHbp occurs post-transcriptionally. **(A)** *N*. *meningitidis* MC58 was grown at the temperatures indicated until mid-log, then subjected to flow cytometry analysis using anti- V1.1 fHbp pAbs to detect surface localised fHbp. Representative graph of fHbp detection at indicated temperatures. Secondary antibody only used as a control (grey). Geometric mean fluorescence of surface expressed fHbp from three independent experiments; error bars are ± SEM. Stastically significant levels of fHbp were detected between bacteria grown at 30°C and 42°C. **(B)** RNA was obtained at mid-log phase from bacteria grown at the temperatures indicated, and samples (20 μg) were separated on agarose-formaldehyde gel and subjected to Northern blot analysis with probes for *fHbp* and *tmRNA*. The position of bands corresponding to the mono- and bi-cistronic transcripts are shown. **(C)** Proteins extracted from the same cultures as in B were analysed by Western blot. Relative levels of RNA and protein are indicated. **(D)** Western blot analysis of protein stability. Bacteria were grown at 30°C or 37°C until mid-log phase, then protein synthesis blocked by the addition of spectinomycin. Samples were removed at time-points afterwards and subjected Western blot analysis. Membranes were probed with antibodies recognising fHbp or RecA (loading control).

We have shown previously that levels of fHbp in the meningococcus increase following growth at higher temperatures [19]. To determine whether this affects the level of fHbp on the bacterial surface, we analysed bacteria grown at different temperatures by flow cytometry using αfHbp pAbs (Fig 1A). The results demonstrate that a rise in temperature is associated with a significant increase in the amount of fHbp on the meningococcus between bacteria grown at 30°C and 42°C.

To establish whether this change is mediated by an alteration in the transcription of *fHbp*, *N*. *meningitidis* was grown to mid-log phase at 30°C, 37°C or 42°C, and *fHbp* mRNA detected by Northern analysis. Of note, levels of the bi- and mono-cistronic transcripts of *fHbp* mRNA were unaffected by temperature, demonstrated by Northern blot analysis (Fig 1B). However, analysis of samples from the same experiment demonstrate that there was an accompanying clear increase in fHbp levels at higher temperatures (Fig 1C) as described previously [19], indicating that thermal regulation of fHbp occurs at the post-transcriptional level.

To examine whether the increase in fHbp at higher temperatures results from an alteration in protein stability, *N*. *meningitidis* was grown at different temperatures to mid-log phase then *de novo* protein synthesis was inhibited by adding spectinomycin to cultures. Western blot analyses of samples taken at times afterwards demonstrate that there is no detectable difference in the stability of fHbp in *N*. *meningitidis* at 30 and 37°C (Fig 1D). Taken together, these results indicate that the thermoregulation of fHbp does not result from a change in transcription or protein degradation.

### The promoter of the monocistronic *fHbp* mRNA is dispensible for thermoregulation

**Fig 2:**
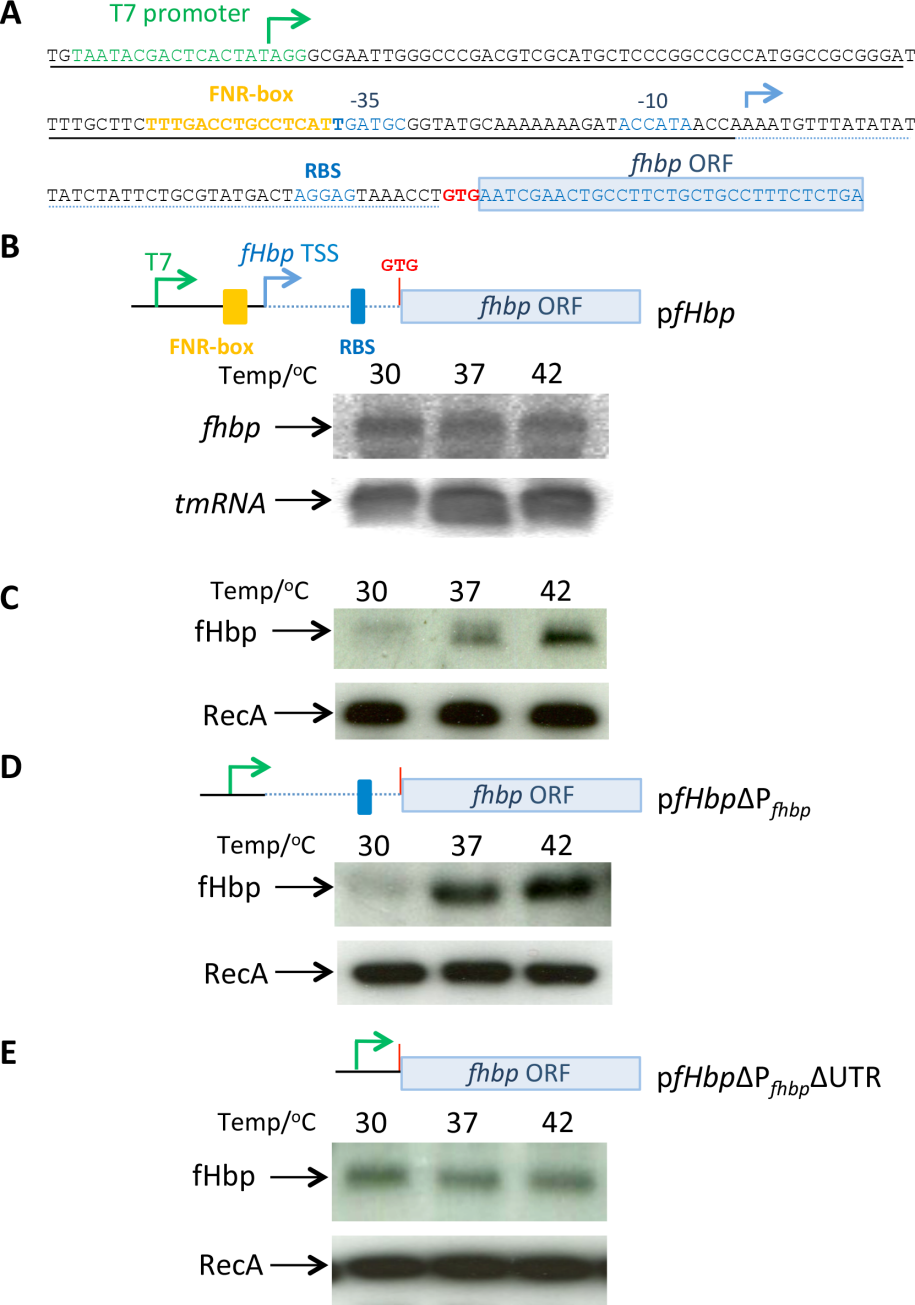
fHbp thermoregulation requires the 5´-UTR of *fHbp* mRNA. **(A)** Sequence of the insert in p*fHbp* indicating the sites of T7 and P_*fHbp*_ promoters (green and blue arrows, respectively), the FNR box (yellow), the P_*fHbp*_ -35 and -10 sequences with the RBS (shown blue), and the start codon, red, GTG). **(B)** *E*. *coli* containing p*fHbp* was grown at the temperatures indicated until mid-log, then RNA was extracted and subjected to Northern analysis with probes for *fHbp* and *tmRNA*. **(C)** Protein was obtained from the same samples as for RNA analysis and examined by Western blot using antibodies against fHbp and RecA (as a loading control). **(D)** *E*. *coli* containing a plasmid (p*fHbp*ΔP_*fHbp*_, deleted sequences underlined with dashed line in panel A) lacking the FNR box and P_*fhbp*_ still demonstrates fHbp thermoregulation. **(E)** Removal of the 5´-UTR from p*fHbp*ΔP_*fHbp*_ (generating p*fHbpΔ*P_*fHbp*_ΔUTR, further deleted sequences underlined with dotted line in panel A) abolishes fHbp thermoregulation.

Thermoregulation of fHbp was analysed in *Escherichia coli* using a plasmid (p*fHbp*) containing the mono-cistronic *fHbp* and its promoter, P_*fHbp*_ (Fig 2A and [19]); results demonstrate that the bi-cistronic mRNA is dispensable for fHbp thermoregulation. To further define the mechanism of thermoregulation, we examined the level of *fHbp* mRNA in *E*. *coli* harbouring p*fHbp* grown at 30°C, 37°C and 42°C; consistent with results obtained with *N*. *meningitidis*, there was no discernible change in transcript levels following growth at different temperatures (Fig 2B), even though fHbp was subject to clear thermoregulation in the same samples (Fig 2C). To identify sequences that contribute to thermoregulation, we generated a plasmid (p*fHbp*ΔP_*fHbp*_) that includes the 5´-UTR of the transcript but lacks its native promoter, P_*fHbp*_; in this plasmid, transcription of *fHbp* occurs from the T7 promoter in the vector (Fig 2A) or the SP6 promoter in the opposite orientation. Again a temperature-dependent increase in fHbp levels was observed in *E*. *coli* harbouring *fHbp* in either orientation in this plasmid (Fig 2D and S1 Fig), demonstrating that P_*fHbp*_ is not required for fHbp thermoregulation. In contrast, deletion of the 5´ UTR from p*fHbp*ΔP_*fHbp*_ (generating p*fHbp*ΔP_*fhbp*_ΔUTR) led to loss of fHbp thermoregulation, indicating that the 5´-UTR of the monocistronic *fHbp* mRNA is required for thermoregulation in this system lacking the bicistronic transcript (Fig 2E).

**Fig 3:**
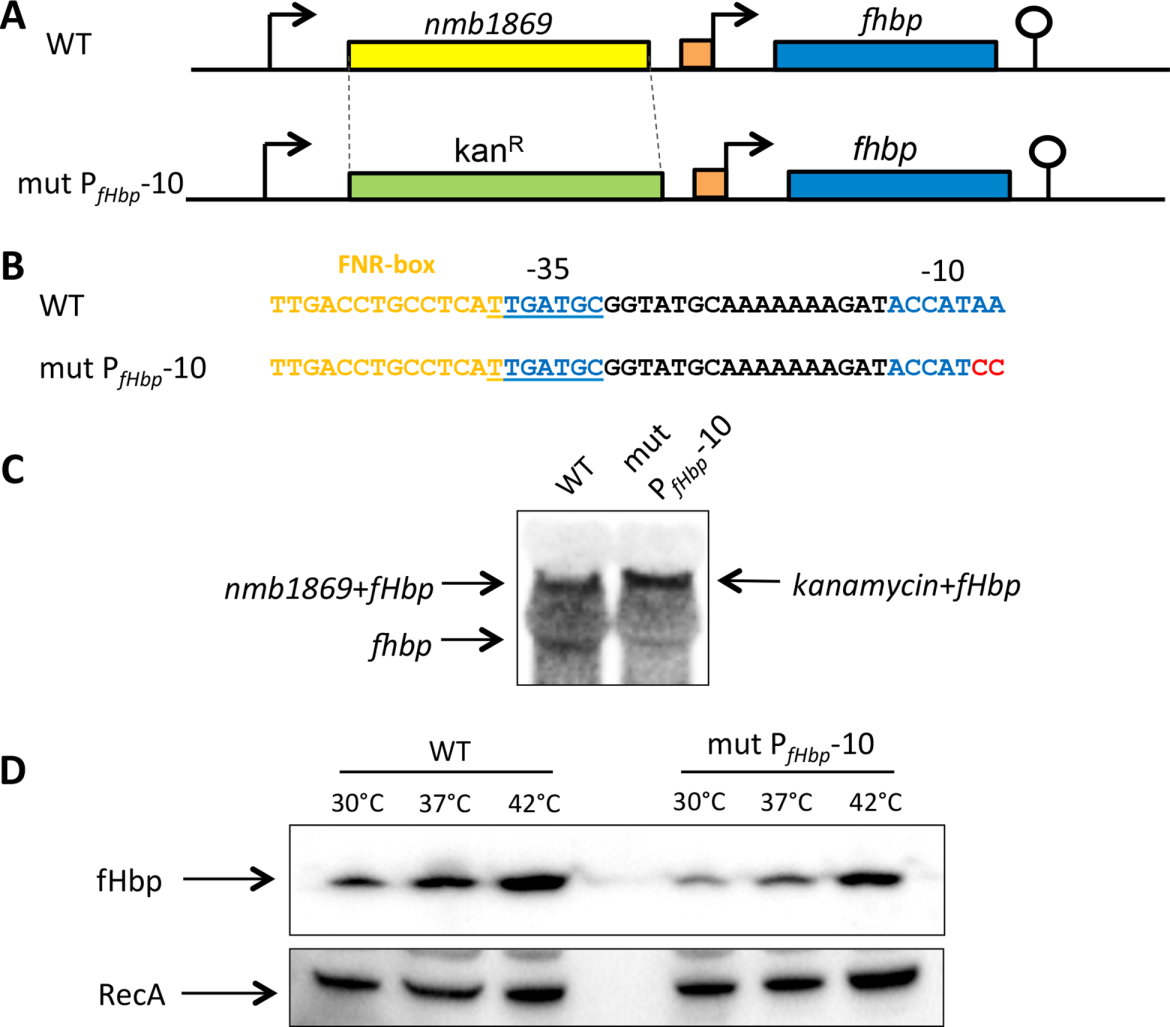
P_*fHbp*_ is dispensable for fHbp thermoregulation in *N*. *meningitidis*. (**A, B**) A *N*. *meningitidis* strain (mut P_*fHbp*_-10) was generated in which *nmb1869* was replaced with a promoterless kanamycin resistance cassette and the -10 sequence of P_*fHbp*_ was mutated (nt. changes shown in red); the position of the predicted -35 and -10 sequences of P_*fHbp*_ are in blue. (**C**) Northern blot analysis confirmed the absence of the monocistronic *fHbp* mRNA in *N*. *meningitidis* mut P_*fHbp*_-10. (**D**) Bacteria were grown in BHI medium until mid-log phase at indicated temperatures, and western blot analysis was performed with antibodies recognising fHbp or RecA (loading control).

To examine the relevance of these findings in *N*. *meningitidis*, we substituted the open reading frame (ORF) upstream of fHbp (*i*.*e*. *nmb1869*, 1065 bp in length) with the 1120 bp kanamycin resistance marker in *N*. *meningitidis* to i) leave the promoter for the bi-cistronic transcript intact, and ii) disrupt P_*fhbp*_ promoter (Fig 3A and 3B), generating strain mutP_*fHbp*_-10. Northern analysis confirmed loss of the monocistronic fHbp mRNA (Fig 3C), and thermoregulation was still observed in the meningococcus with the bi-cistronic transcript alone (Fig 3D). Together with the findings in *E*. *coli* (Fig 2), the results provide evidence that the monocistronic P_*fHbp*_ is dispensable for fHbp thermoregulation.

### Expression of fHbp is influenced by sequences in the coding region

**Fig 4:**
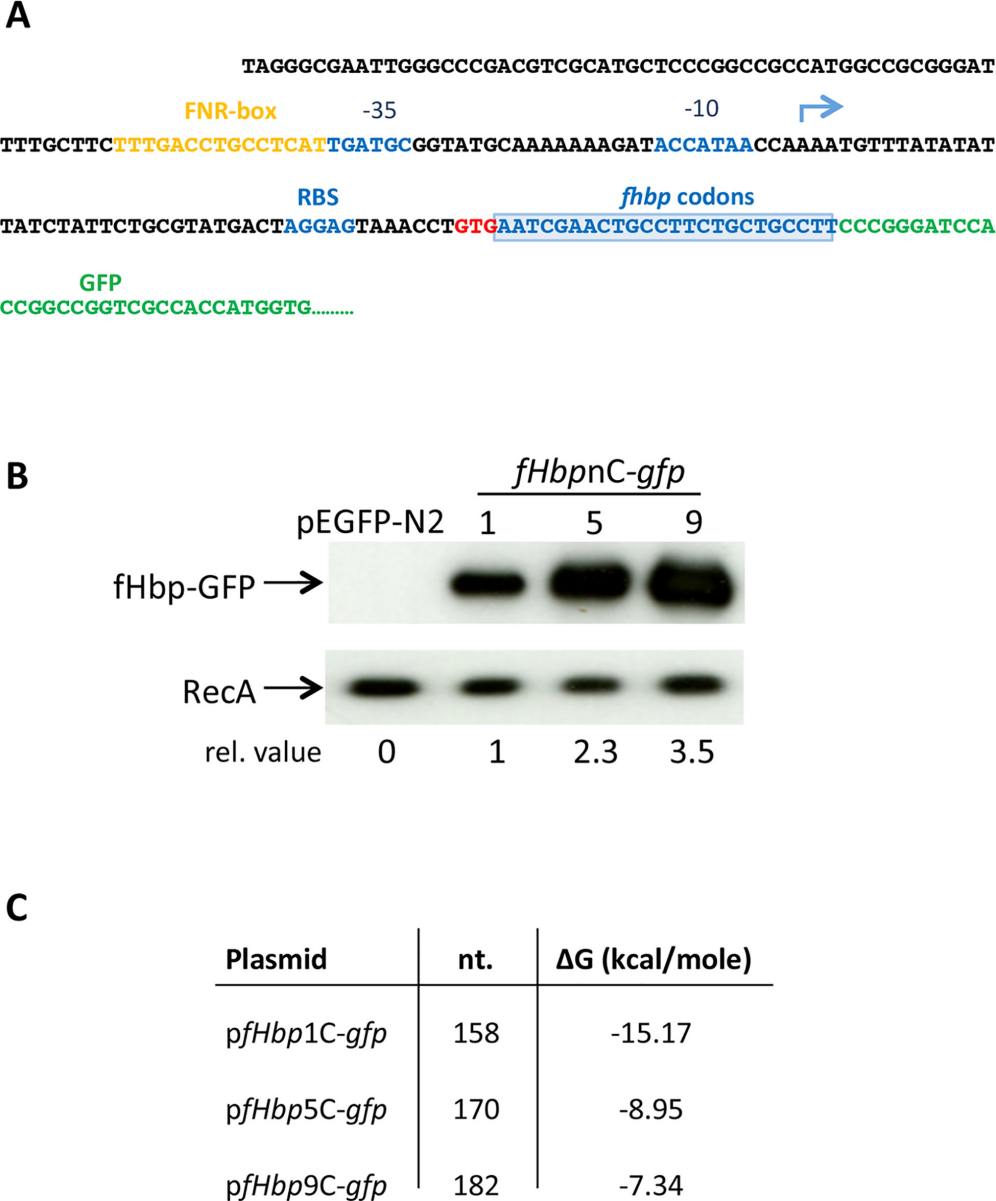
Translation of fHbp is enhanced by its initial codons. **(A)** Sequence of p*fHbp-*9C*-gfp*, a GFP reporter plasmid including nine *fHbp* codons. The construct includes P_*fHbp*_ (-10 and -35 indicated, and blue arrow for the TSS) with the 5´-UTR and nine codons of *fHbp* open as a translational fusion with GFP in pEGFP-N_2_: P_*fHbp*_, blue arrow; blue box denotes the first nine codons of *fHbp*. (**B)** Cultures of *E*. *coli* containing plasmids harbouring one, five or nine codons, or containing the empty vector pEGFP-N_2_ were grown at 37°C until mid-log phase. Whole cell lysates were subjected to Western blot analysis with antibodies recognising GFP or RecA (loading control). Relative expression values (indicated). **(C)** Table of the predicted minimum free energies in kcal/mole (ΔG) of potential secondary RNA structures of sequences introduced into the plasmids (A) with the number of nucleotides shown (nt.).

Next we generated plasmids containing the 5´-UTR with the first one, five or nine codons of *fHbp* fused to GFP as a reporter (p*fHbp*1C-*gfp*, p*fHbp*5C-*gfp* and p*fHbp*9C-*gfp*, respectively, Fig 4A); we were unable to generate constructs with more codons, probably because of toxic effects of the fHbp leader sequence in *E*. *coli*. The plasmids lack a T7 promoter [22], so expression is governed by the native *fHbp* promoter. We observed a clear correlation between number of *fHbp* codons in the plasmid and levels of the GFP reporter, with approximately 3.5-fold higher GFP in bacteria harbouring p*fHbp*_*9C*_ *-gfp* compared with *fHbp*_*1C*_ *-gfp* following growth at 37°C (Fig 4B). The increase in GFP levels observed with p*fHbp*_*9C*_ *-gfp* is not caused by a change in protein or RNA stability (S2 Fig), but instead correlates with the predicted minimum free energy values (using the Vienna *RNAfold* package, Fig 4C) and putative secondary structures (S3 Fig).

### Gradual thermoregulation of fHbp in an *in vitro* system

**Fig 5:**
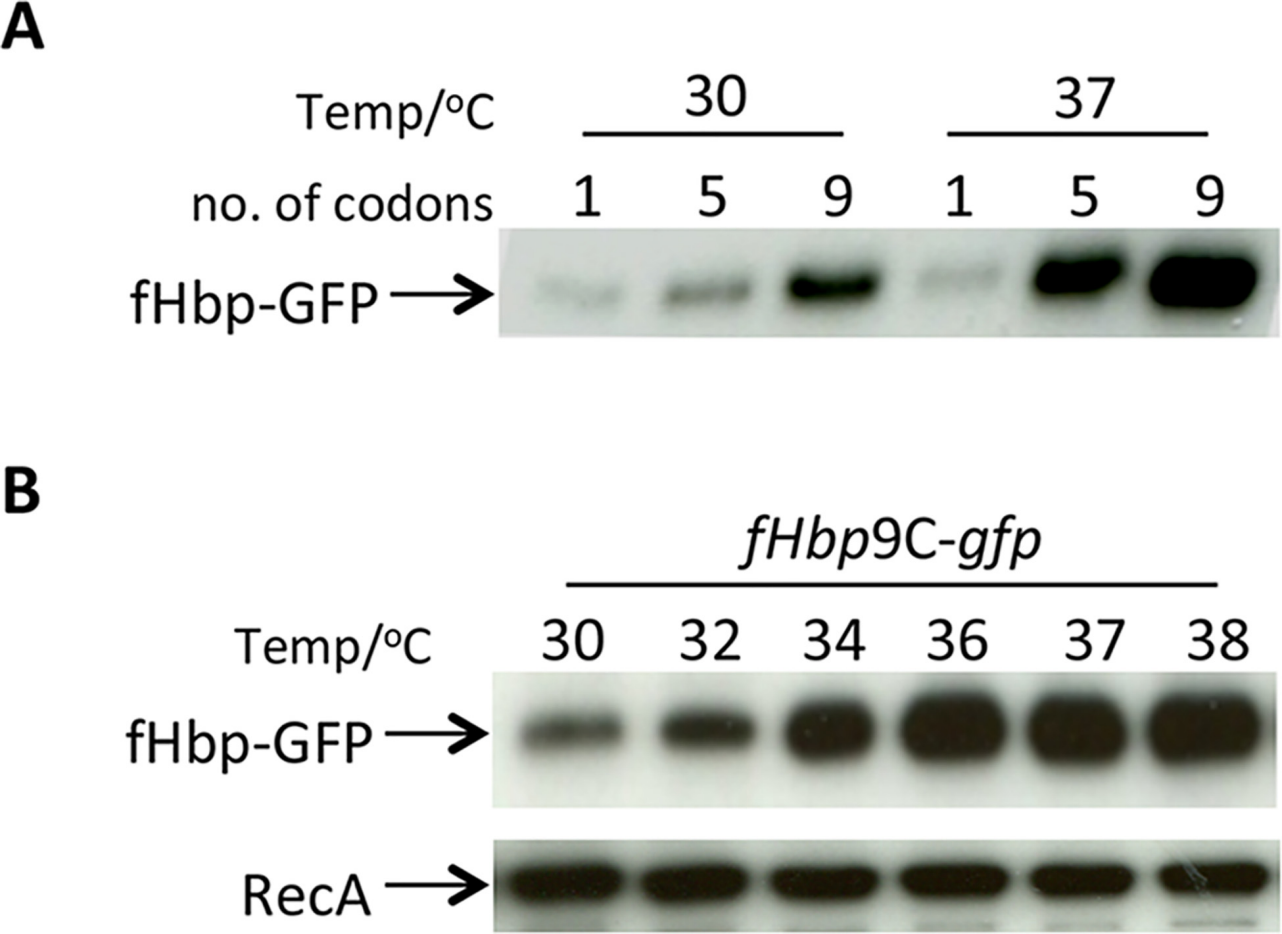
Gradual thermoregulation of fHbp-GFP. **(A)** *In vitro* transcription/translation assays using DNA from p*fHbp-*1C*-gfp* p*fHbp-*5C*-gfp* or p*fHbp-*9C*-gfp* (number of codons shown above each lane) performed at the indicated temperatures for 1 hr. Samples were subject to Western analysis. (B) Gradual thermoregulation of fHbp-GFP. RNA was isolated from *E*. *coli* containing p*fHbp-*9C*-gfp* construct was subjected to *in vitro* translation at indicated temperatures. Western analysis was performed using antibodies recognising GFP or RecA.

To determine whether thermoregulation of fHbp was also observed in a cell free system, *in vitro* transcription/translation assays were performed with p*fHbp*1C-*gfp*, p*fHbp*5C-*gfp* and p*fHbp*9C-*gfp*. DNA from these plasmids was transcribed and translated in a continuous manner at 30°C or 37°C, and GFP production assayed by Western blot analysis (Fig 5A). There was increased GFP generated in assays performed at the higher temperature with p*fHbp*5C-*gfp* and p*fHbp*9C-*gfp*; however thermoregulation was not observed in assays containing p*fHbp*1C-*gfp*. Overall levels of GFP produced were higher in assays with plasmids harbouring more codons, consistent with sequences in the ORF enhancing translation efficiency. Therefore, the fHbp_9C_-GFP construct was used in subsequent experiments.

To further examine the dynamics of fHbp thermoregulation, *in vitro* transcription/translation reactions were conducted with p*fHbp*9C-*gfp* at temperatures between 30°C and 38°C. Equal amounts of plasmid were used as the substrate for *in vitro* transcription/translation reactions performed at 30°C, 34°C, 36°C, 37°C and 38°C for one hour, and reaction products were analysed by Western blotting. The results demonstrate that levels of the GFP reporter rise gradually in response to increasing temperature over a physiological range (Fig 5B) similar to the *cssA* thermosensor [19].

### Anti-ribosomal binding site sequences in the *fHbp* ORF contribute to fHbp thermosensing

**Fig 6:**
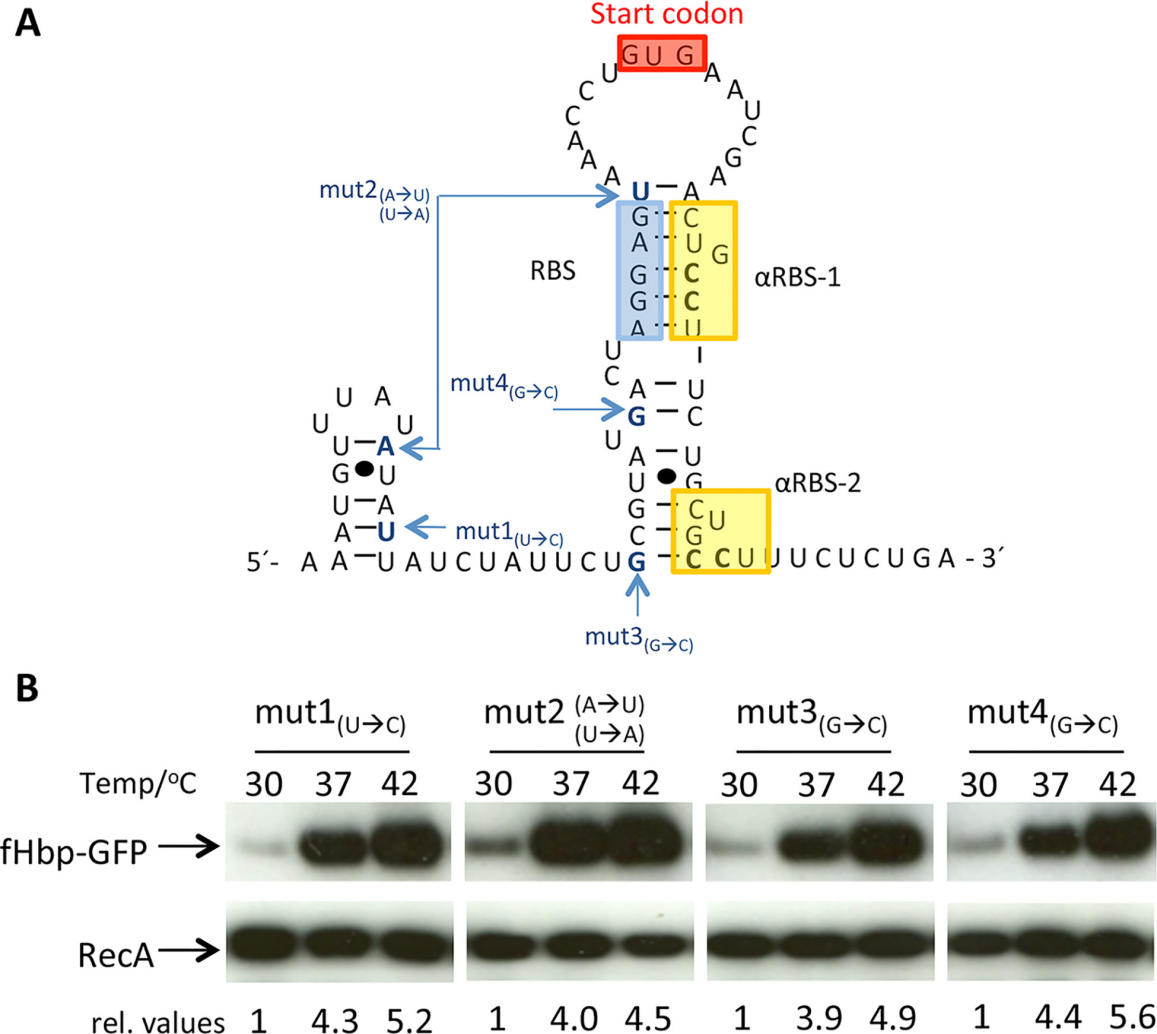
Modification of nucleotides in the *fHbp* 5´-UTR does not abolish thermoregulation. **(A)** Predicted RNA secondary structure of the whole 5´-UTR and 34 bases of *fHbp*-mRNA. (RBS indicated in blue, start codon in red, putative α-RBSs in yellow). The nucleotides modified in p*fHbp-*9C*-gfp* are indicated. **(B)** Western blot analysis of fHbp-GFP levels in *E*. *coli* harbouring plasmids with modified *fHbp* 5´-UTRs. Bacteria were grown to mid-log phase at the indicated temperatures, and whole cell lysates subjected to Western blot analysis with antibodies recognising GFP or RecA. Relative values (rel. values) of GFP are indicated below each lane.

Next, we performed mutagenesis to identify key nucleotides involved in the formation of putative RNA secondary structures in the *fHbp* mRNA that mediate thermoregulation. Several point mutations were introduced upstream of the *fHbp* GTG start codon in p*fHbp*9C-*gfp* (Fig 6A), and their effect examined during growth of *E*. *coli* at 30°C, 37°C and 42°C. Proteins were isolated from the bacteria and levels of GFP expression examined by Western blot analysis. However, none of the single base substitutions in the 5´-UTR disrupted thermoregulation (Fig 6B), indicating that these changes are insufficient to perturb RNA secondary structures that are necessary for thermosensing.

**Fig 7:**
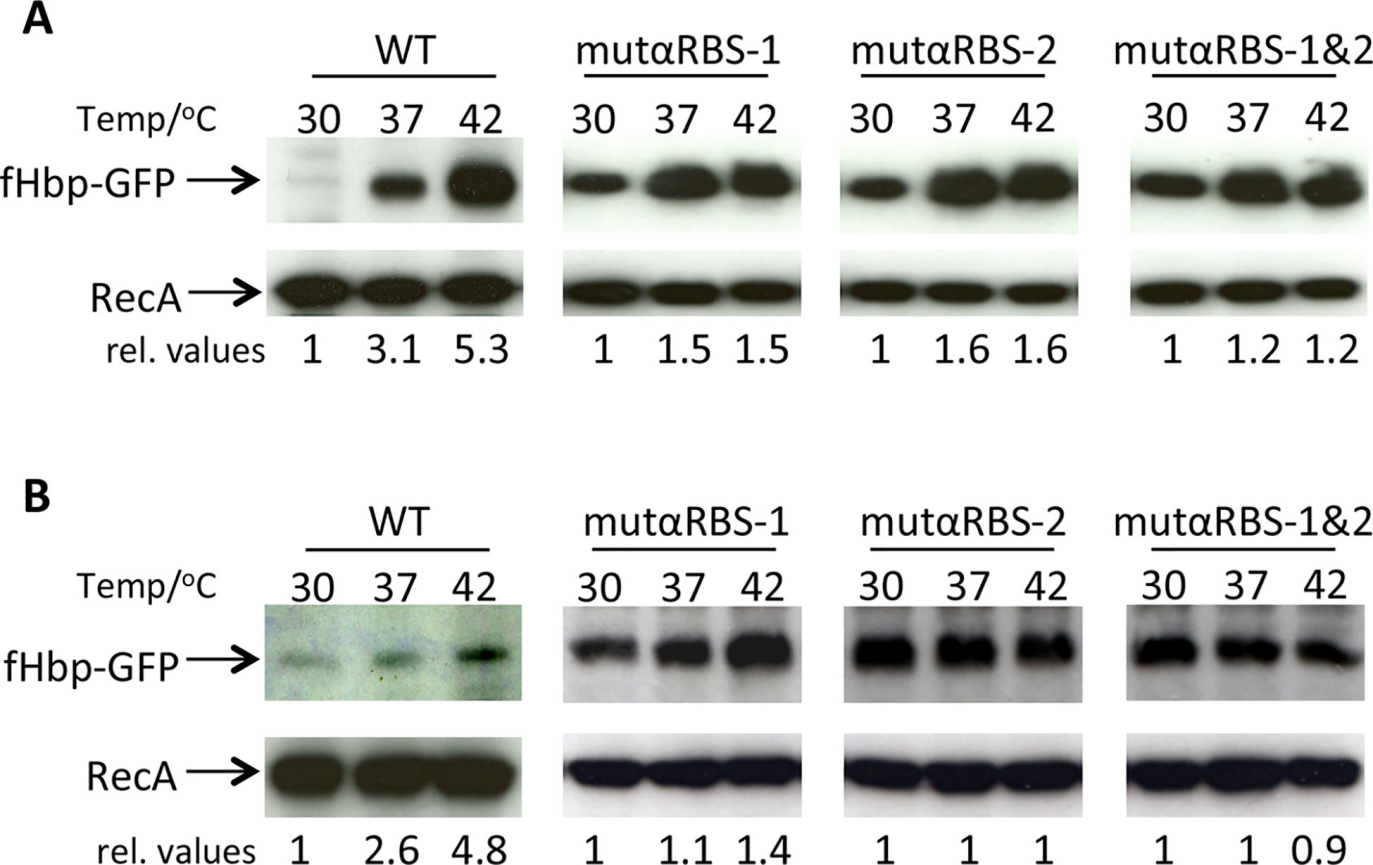
αRBS sequences in the open reading frame are necessary for fHbp thermoregulation. **(A)** Western blot analysis of fHbp-GFP levels *in E*. *coli* harbouring p*fHbp-*9C*-gfp* (WT) or the plasmid with modified αRBS-1, αRBS-2 or both (αRBS-1&2). Bacteria were grown at the temperatures indicated to mid-log phase, and cell lysates were subjected to Western blot analysis. The membranes were incubated with antibodies recognising fHbp or RecA. **(B)** *In vitro* translation of RNA from *E*. *coli* harbouring p*fHbp-*9C*-gfp* or plasmids containing altered RBSs (indicated). *in vitro* translation was performed at the temperatures indicated and subject to Western analysis with antibodies recognising GFP and RecA (loading control). mut-αRBS-1 and mut-αRBS-2 showed reduced thermosensing ability whereas modification of both αRBS-1 and 2 (mut-αRBS-1&2) almost completely abolishes thermoregulation both in *E*. *coli* and in *in vitro* translation assays. Relative values (rel. values) of GFP are indicated below each lane.

On further inspection, we identified two copies of a 6 bp repeat (CUGCCU) within the *fHbp* coding region, close to the start codon and potentially able to base-pair with the ribosome binding site (RBS); we termed these repeats αRBS-1 and αRBS-2 (Fig 6A). We reasoned that the two cytosines could base-pair with the guanines in the RBS (AGGAG) and prevent ribosomes binding to the nascent transcript. Therefore, site-directed mutagenesis was performed to modify the two cytosines to guanines in each αRBS (*i*.*e*. CUGCCU to CUGGGU) either singly (generating mutαRBS-1 and mutαRBS-2), or together (mutαRBS-1/2). Constructs were introduced into *E*. *coli*, which was then grown at 30°C, 37°C and 42°C, and the level of GFP examined by Western blot analysis. All three constructs displayed reduced *fHbp* thermoregulation when compared to the plasmid containing wild-type sequences. Comparing cultures grown at 30°C or 42°C, the level of GFP increased by 1.5-fold and 1.6-fold for cells harbouring mutαRBS-1 and mutαRBS-2, respectively, whereas cells containing mutαRBS-1/2, showed a 1.2-fold increase (Fig 7A). In contrast, GFP levels increased by 5.3-fold with the wild-type sequence in the plasmid. RecA levels were stable in all conditions, and modification of the αRBS sequences did not affect the stability of the mRNA (S4 Fig). We also introduced the αRBS mutations into p*fHbp*, and found that modifications impaired the thermoregulation of fHbp (S5 Fig), although changing the fHbp sequence affected its detection by western blot analysis. It is likely that the lack of detection of fHbp resulted in changes from the protein sequence (S5 Fig); mutαRBS-1 results in an Ala to Gly substitution, while mutαRBS-2 alters a Cys to Trp, and a Leu to Val (S5 Fig).

Next we examined whether the α-RBS affect the translation of fHbp. *in vitro* translation reactions were performed with equal amounts of total cellular RNA extracted from *E*. *coli* strains harbouring the different constructs, and assays conducted at 30°C, 37°C or 42°C. The *in vitro* synthesised proteins were extracted and assayed by Western blot analysis. The wild-type construct showed thermoregulation, whereas the three constructs with altered α-RBS did not display thermoregulation (Fig 7B), similar to results found in *E*. *coli*; RecA levels were the same for all conditions examined. Taken together, these results demonstrate that both α-RBS sequences contribute to fHbp thermosensing. Additionally, we attempted to introduce the α-RBS mutations into *N*. *meningitidis* either under the control of the mono-cistronic or bi-cistronic promoter (S6 Fig). However similar to results in *E*. *coli* (S5 Fig), fHbp was undetectable by Western blot analysis of cell lysates of the meningococcus containing mutαRBS-1/2, presumably because the amino acid changes affected the processing of the N-terminal region of fHbp and affected its stability. We considered introducing single C to G mutations into α-RBS1 and/or α-RBS2, but these nucleotide changes of the α-RBSs are not predicted to impact the overall structure of *fHbp* mRNA (S7 Fig).

## Discussion

The meningococcus has successfully evolved to survive in the human nasopharynx, which is its only natural habitat. Colonisation with this bacterium is frequent in young adults (up to 70% among those living in institutionalised settings such as prisons and barracks) [1], and a single strain can persist in the same individual for months in the upper airway [23]. Within this environment, there are several temperature gradients. For example, the temperature on the surface of the anterior nares is around 30°C at the end of inspiration, and rises to around 34°C in the posterior nasopharynx and tonsillar region [13]. Both these sites on the mucosal surface are significantly cooler than the core body temperature of 37°C, where the bacterium replicates during invasive disease. Additionally, fluctuations in the local temperature will be generated by acute inflammation (resulting in increased blood flow) and systemic illnesses, such as influenza, which provoke a febrile response [24].

We have shown previously that temperature is an important environmental cue for the meningococcus [19]. The production of fHbp and enzymes necessary for capsule biosynthesis and LPS sialylation are increased as the temperature rises from 30°C through to 42°C. The enhancement of immune evasion by the bacterium may reflect transition from the cool anterior portion of the nasopharynx (lined by keratinised squamous cells with little lymphoid tissue) to the warmer posterior nasopharynx, where the meningococcus is found in tonsillar tissue [16]. Furthermore, increased temperature could act as a signal of local inflammation [18] or transition to the sub-mucosal layer, where the bacterium will be exposed to immune effectors.

A notable feature of the meningococcus is its capacity to evade exclusion by the human immune system [4]. Surface structures expressed by *N*. *meningitidis*, including Type IV pili, undergo frequent antigenic variation in certain strains [25], while the serogroup B capsule is a molecular mimic of a human post-translational modification [26]. Additionally, fHbp mediates high affinity interactions with the complement regulator, CFH using ligand mimicry [8], and a CFH antagonist CFHR3 [9].

We have shown previously that the biosynthesis of sialic acid-containing capsules is governed by an RNA thermosensor in *N*. *meningitidis* [19]. A fundamental advantage of RNA thermosensors is that they operate at the post-transcriptional level [20], so do not require a dedicated sensing pathway or *de novo* transcription to exert their effect. Therefore, RNA thermosensors are an energetically efficient strategy that offers rapid responses to abrupt changes in the temperature, as seen during the onset of inflammation.

For many bacteria, an increase in temperature is a key signal during their acquisition and ingestion by a mammalian host. For example, in enteric pathogens, RNA thermosensors are known to modulate expression of a transcriptional regulator, which in turn orchestrates the expression of a suite of genes that enable the pathogen to survive in its new environment [20]. Examples include the RNA thermosensors in *prfA* in *Listeria monocytogenes* [22], *agsA* in *Salmonella* [27], and *lcrF* in *Yersinia* spp. [28,29]. Thermosensors also govern iron acquisition systems in *Shigella* and *E*. *coli* [30], and *Vibrio cholerae* [31]. We found that the *prfA* thermosensor operates as an ON:OFF switch [32], similar to an RNA thermosensor in Cyanobacteria [33]. The abrupt thermodynamic response of the *prfA* thermosensor is consistent with *Listeria* undergoing large temperature fluctuations during its transition from the external environment to the intestinal tract. However, our *in vitro* transcription/translation assays reveal that the *fHbp* thermosensor exhibits a gradual change over a range of physiologically relevant temperatures *i*.*e*. 32–35°C [17], similar to the *N*. *meningitidis* capsule thermosensor [19]. The careful calibration of responses to temperature may be a feature of thermosensors in microbes that exist in close association with thermal gradients within hosts, particularly in the upper airway.

Previous work demonstrated that the expression of fHbp is mediated by bi-cistronic and mono-cistronic transcripts, with the shorter mono-cistronic *fHbp* transcript controlled by the global regulator of anaerobic metabolism, FNR [21]. Several lines of evidence demonstrate that the mono-cistronic transcript is sufficient but not necessary for fHbp thermoregulation, which is also detected with the bi-cistronic transcript in *N*. *meningitidis*. Thermoregulation was detected with the mono-cistronic transcript alone in *E*. *coli* and using *in vitro* transcription/translation assays. Additionally we generated a *N*. *meningitidis* mutant lacking the bi-cistronic transcript (by inserting a kanamycin resistance cassette with a terminator upstream of P_*fHbp*_). Even though fHbp levels are lower than in the wild-type strain, consistent with previous work [21], the strain still exhibited thermoregulation of fHbp (S6 Fig).

The fHbp transcript is not predicted to contain a ROSE element by RNA structure predictions and does not contain a U(U/C)GCU sequence close to the RBS, which is often present in this class of thermosensors [20]. Furthermore, the potential involvement of a temperature regulated sRNA is unlikely as fHbp thermoregulation occurs both in *E*. *coli* and *in vitro* assays. Instead, our experiments indicate that the mechanism responsible for fHbp thermoregulation depends on two α-RBS sequences located in the *fHbp* ORF. Modification of each α-RBS independently reduced thermoregulation, which was virtually abolished when both α-RBSs were altered, indicating some redundancy in their function. In contrast, mutation of the *fHbp* 5´-UTR had little or no effect on thermoregulation. Therefore our results support the model in which the fHbp transcript forms a stem loop at lower temperatures with the RBS occluded by base pairing with one of two α-RBS sequences located in the ORF. Attempts were also made to introduce the α-RBS mutations into *N*. *meningitidis*. However, we could not detect fHbp in the meningococcus containing modified α-RBSs (S6 Fig), even though fHbp was detected in *E*. *coli* using equivalent constructs on a multi-copy plasmid. The nucleotide changes lead to an alteration in the amino acid sequence of fHbp which might affect lipoprotein localisation and processing, which is distinct in *N*. *meningitidis* and *E*. *coli* [34]. We considered other modifications to the αRBS sequences. However the wobble rule of RNA:RNA binding [35] means that G can bind several bases (*e*.*g*. U and A), so their impact on the secondary structure of the RNA would have been uncertain. The precise contribution of the individual α-RBS sequences to the *fHbp* RNA thermosensor will be determined in future structural studies.

We also found that the ORF of *fHbp* contributes to levels of the protein in the meningococcus. Inclusion of first nine codons of fHbp in GFP fusions resulted in significantly higher protein levels of reporter the compared with constructs containing less sequence. This was not mediated by changes in protein stability (S2A Fig), and could result from increased efficiency of translation although these findings need to be confirmed in *N*. *meningitidis*.

Of note, attempts were made to generate GFP fusions with 12 and 20 fHbp codons in *E*. *coli* to define sequences required for maximal fHbp expression. However, it was not possible to obtain the *fHbp*_*12C*_ *-gfp* and *fHbp*_*20C*_ *-gfp* constructs. fHbp is a surface lipoprotein bound to the outer membrane via an N-terminal lipid anchor [10,11]. Unlike in the meningococcus, we have been unable to detect fHbp on the surface of *E*. *coli* even when expressing the full length protein, demonstrating that there are differences in protein sorting in these two Gram negative bacteria. It is therefore possible that additional *fHbp* sequences in *gfp* fusions cause accumulation of GFP at aberrant cellular sites, impairing bacterial viability.

fHbp thermoregulation may have implications for the effect of vaccines that target fHbp. Levels of this vaccine antigen in the meningococcus in the upper airway (at 32–35°C) may be lower than in assays to assess immune responses within the laboratory, which are typically performed at 37°C [36,37]. Therefore, vaccines that include fHbp might not impose selective pressure on bacteria at the mucosal surface in the upper airway, and offer limited herd immunity [38]. This is consistent with a recent study demonstrating that immunisation with a vaccine containing fHbp has a limited impact on the acquisition of meningococcal carriage among university students [39].

Aside from factors involved in immune evasion, RNA thermometers may control other features in *N*. *meningitidis* and other bacteria that inhabit the nasopharynx. Previous studies have focused mainly on the effect of temperature on mRNA levels in the meningococcus [40]. The identification of further RNA thermosensors will require bio-informatic and proteomic approaches. Further understanding the mechanisms of thermoregulation could be informative about strategies of immune evasion employed by this important pathogen, its adhesive properties, and the acquisition of relevant nutrients at different sites in the upper airways [41].

## Methods

### Strains, plasmids and culture conditions

*N*. *meningitidis* was grown in Brain Heart Infusion broth (BHI, Oxoid, 37 g dissolved in 1 L dH_2_O with 1 g starch) or on BHI agar (1.5% w/v) supplemented with 5% Levinthal’s base (500 ml defibrinated horse blood, autoclaved with 1 L BHI broth). Bacteria on solid media were incubated for 16–18 hours at 37˚C with 5% CO_2_. Liquid cultures (10 ml) were inoculated with 10^9^ bacteria and grown at 37˚C with shaking (180 r.p.m.) to an optical density (O.D.) measured at 600 nm of ~0.5 unless otherwise stated.

*E*. *coli* was grown in Luria-Bertani (LB) broth (2% w/v in dH_2_O, Oxoid, UK) or on LB agar (1% w/v) plates. Liquid cultures of *E*. *coli* were grown in 4 ml of media inoculated from a single colony overnight at 37˚C with shaking (180 r.p.m.). Overnight grown bacteria were diluted 1 in 100 in media and grown to an Optical Density (OD) A_600_ of ~0.5. When necessary antibiotics were added to the following final concentrations: carbenicillin, 100 μg ml^-1^; kanamycin, 50 μg ml^-1^; rifampicin, 250 μg ml^-1^. The strains and plasmids used in this study are listed in Table 1.

**Table 1:** Plasmids used in this study.

Name	Description/Sequence	Reference
pGEM-T	Vector for translational fusions, Amp^R^	Promega
pEGFP-N_2_	GFP Translational fusions, Kan^R^	Clontech
pfHbp	fHbp clone in pGEM-T, Amp^R^	This study
pfHbpΔp+TSS	fHbp clone in pGEM-T, without native promoter. Amp^R^	This study
pfHbpΔp+ΔTSS-T7	fHbp clone in pGEM-T, downstream of T7 promoter, without native promoter and transcriptional start site. Amp^R^	This study
pfHbpΔp+ΔTSS-SP6	fHbp clone in pGEM-T, downstream of SP6 promoter, without native promoter and transcriptional start site. Amp^R^	This study
pPilE	PilE clone in pGEM-T, Amp^R^	This study
fHbp_1C_-GFP	fHbp 5´-UTR with 1 codon clone in pEGFP-N_2_. Kan^R^	This study
fHbp_5C_-GFP	fHbp 5´-UTR with 5 codon clone in pEGFP-N_2_. Kan^R^	This study
fHbp_9C_-GFP	fHbp 5´-UTR with 9 codon clone in pEGFP-N_2_. Kan^R^	This study
fHbp_9C_-GFP-Mut1	fHbp 5´-UTR with 9 codon clone in pEGFP-N_2_ with Mut1. Kan^R^	This study
fHbp_9C_-GFP-Mut2	fHbp 5´-UTR with 9 codon clone in pEGFP-N_2_ with Mut2. Kan^R^	This study
fHbp_9C_-GFP-Mut3	fHbp 5´-UTR with 9 codon clone in pEGFP-N_2_ with Mut3. Kan^R^	This study
fHbp_9C_-GFP-Mut4	fHbp 5´-UTR with 9 codon clone in pEGFP-N_2_ with Mut4. Kan^R^	This study
fHbp_9C_-GFP-Mut-αRBS-1	fHbp 5´-UTR with 9 codon clone in pEGFP-N_2_ with MutαRBS-1. Kan^R^	This study
fHbp_9C_-GFP-Mut-αRBS-2	fHbp 5´-UTR with 9 codon clone in pEGFP-N_2_ with MutαRBS-2. Kan^R^	This study
fHbp_9C_-GFP-Mut-αRBS-1&2	fHbp 5´-UTR with 9 codon clone in pEGFP-N_2_ with MutαRBS-1&2. Kan^R^	This study
nmb1869+ fHbp_9C_-GFP	Nmb1869+ fHbp 5´-UTR with 9 codon clone in pEGFP-N_2_. Kan^R^	This study
Nmb1869-KM-fHbp	Nmb1869+Kan+fHBP clone in pGEM-T for mutational study in *Nm*. (NcoI and NotI digestion)	This study

### Flow cytometry

*N*. *meningitidis* was grown in liquid culture to mid. log phase at 30°C, 37°C or 42°C, prior to fixation for two hours in 3% paraformaldehyde. Surface localisation of fHbp on *N*. *meningitidis* was detected using anti-fHbp V1.1 pAbs and goat anti-mouse IgG-Alexa Fluor 647 conjugate (Molecular Probes, LifeTechnologies). Samples were run on a FACSCalibur (BD Biosciences), and at least 10^4^ events recorded before results were analysed by calculating the geometric mean fluorescence intensity in FlowJo vX software (Tree Star).

### Plasmids and construction of *N*. *meningitidis* mutants

A *N*. *meningitidis* strain containing only the monocistronic *fHbp* transcript was generated by insertion of a kanamycin resistance cassette with a Rho-independent terminator upstream of P_*fHbp*_ (S6 Fig). Upstream and downstream fragments, and the kanamycin resistance cassette were amplified by from gDNA using the primer pairs fHbp-KM-(1)-F/fHbp-KM-(1)-R, fHbp-KM-(2)-F/fHbp-KM-(2)-R and fHbp-KM-(3)-F/fHbp-KM-(3)-R, respectively. Details of primers used in this study are given in Table 2. PCR products were ligated into pGEM-T (Promega) following Gibson Assembly (NEB), then digested with *Nco*I and *Not*I (NEB). Transformation of *N*. *meningitidis* strain MC58 was performed as described previously [42].

**Table 2:** Table of primers

Primer name	Sequence	Relevant sites
fHbp(c)-F	GGTTGCCTGTAAACAAAATGC	
fHbp(c)-R	CGGACGGTGCAATACAAAAT	
PPilE-F	GGGGGAATTCCGCGCCTGTCAGATAAACC	
PPilE-R	GGGGCCCGGGTCGATAGGAAATCTACATCC	
fHbp(TTS)-U	AAATGTTTATATATTATCTATTCTGC	
fHbp(SC)-U	GTGAATCGAACTGCCTTCTG	
fHbp-GFP-F	GGGGGAATTCGGTTGCCTGTAAACAAAATGC	*Eco*RI
fHbp-GFP1C-R	GGGGCCCGGGCACAGGTTTACTCCTAGTCATACG	*Sma*I/*Xma*I
fHbp-GFP5C-R	GGGGCCCGGGGGCAGTTCGATTCACAGGTTTACTCC	*Sma*I/*Xma*I
fHbp-GFP9C-R	GGGGCCCGGGAAGGCAGCAGAAGGCAGTTCG	*Sma*I/*Xma*I
nmb1869+ fHbp_9C_-GFP-F	GGGGGAATTCAAACCTGCATACTGGCATCG	*Eco*RI
Mut1-F	CCAAAATGTTTATATACTATCTATTCTGCGTATGACTAGGAGTAAACC	
Mut1-R	GGTTTACTCCTAGTCATACGCAGAATAGATAGTATATAAACATTTTGG	
Mut2-F	CCAAAATGTTTATATATTACCTATTCTGCGTATGACTAGGAGTAAACC	
Mut2-R	GGTTTACTCCTAGTCATACGCAGAATAGGTAATATATAAACATTTTGG	
Mut3-F	CCAAAATGTTTATATTTTATCTATTCTGCGTATGACTAGGAGAAAACC	
Mut3-R	GGTTTTCTCCTAGTCATACGCAGAATAGATAAAATATAAACATTTTGG	
Mut4-F	CCAAAATGTTTATATATTATCTATTCTCCGTATGACTAGGAGTAAACC	
Mut4-R	GGTTTACTCCTAGTCATACGGAGAATAGATAATATATAAACATTTTGG	
mutαRBS-1-F	TGCGTATGACTAGGAGTAAACCTGTGAATCGAACTGGGTTCTGCTGCCTT	
mutαRBS-1-R	AAGGCAGCAGAACCCAGTTCGATTCACAGGTTTACTCCTAGTCATACGCA	
mutαRBS-2-F	TGCGTATGACTAGGAGTAAACCTGTGAATCGAACTGCCTTCTGCTGGGTT	
mutαRBS-2-R	AACCCAGCAGAAGGCAGTTCGATTCACAGGTTTACTCCTAGTCATACGCA	
mutαRBS-2-F	TGCGTATGACTAGGAGTAAACCTGTGAATCGAACTGGGTTCTGCTGGGTT	
mutαRBS-2-R	AACCCAGCAGAACCCAGTTCGATTCACAGGTTTACTCCTAGTCATACGCA	
fHbp-U	AAACGAGAAACTGAAGCTGGC	
fHbp-D	CACTCTCCAAGGTAATGAGC	
tmRNA-U	CGAAACCCAAGGTGCATGC	
tmRNA-D	CAGGGCTTCCACGCG	
pGEM-T easy vector-F	TGTAATACGACTCACTATAGGGCGAATTGGGCCCGACGTCGCATGCTCCCGGCCGCCATGGCCGCGGGAT	
pGEM-T easy vector-R	ATTTAGGTGACACTATAGAATACTCAAGCTATGCATCCAACGCGTTGGGAGCTCTCCCATATGGTCGACCTGCAGGCGGCCGCACTAGTGAT	
Fhbp-KM-(1)-F	CCCGGCCGCCATGGCCGCGGGATGAAGAAATCGTCGAAGGCAT	
Fhbp-KM-(1)-R	CGATGATGGTTGGAATTCACTGGTTCAGACGGCATTTTGTTTACAGG	
Fhbp-KM-(2)-F	CCTGTAAACAAAATGCCGTCTGAACCAGTGAATTCCAACCATCATCG	
Fhbp-KM-(2)-R	AAATCAAATGTCGTCCGAACGGCGGTCGACTCTAGAGGATCCCCGGG	
Fhbp-KM-(3)-F	cccggggatcctctagagtcgaccGCCGTTCGGACGACATTTGATTT	
Fhbp-KM-(3)-R	CCTGCAGGCGGCCGCACTAGTGATCGGACTGATCCAGCGTCAAAGACTGC	
PROM-10-MUT1-F	CCCGGCCGCCATGGCCGCGGGATAGCTTGCGAGCCAGCGTCCGTTCC	
PROM-10-MUT1-R	cgtttcccgttgaatatggctcatTTGTGTCTCCTTGGGCAATAGG	
PROM-10-MUT2-F	CCTATTGCCCAAGGAGACACAAatgagccatattcaacgggaaacg	
PROM-10-MUT2-R	GCATTTTGTTTACAGGCAACCTGttagaaaaactcatcgagcatcaaatg	
PROM-10-MUT3-F	catttgatgctcgatgagtttttctaaCAGGTTGCCTGTAAACAAAATGC	
PROM-10-MUT3-R	CCTAGTCATACGCAGAATAGATAATATATAAACATTTTGGTGGTGGTATC	
PROM-10-MUT4-F	GATACCACCACCAAAATGTTTATATATTATCTATTCTGCGTATGACTAGG	
PROM-10-MUT4-R	CCTTTGTCTTTATGGTCGAGCGGTGCGGTTAGTGCATCGGCAAGCCCCGC	
PROM-10-MUT5-F	GCGGGGCTTGCCGATGCACTAACCGCACCGCTCGACCATAAAGACAAAGG	
PROM-10-MUT5-R	CCTGCAGGCGGCCGCACTAGTGAT TTATTGCTTGGCGGCAAGGCCG	

*N*. *meningitidis* MC58 containing only the bicistronic *fHbp* transcript was generated by replacing the *nmb1869* (the gene upstream of fHbp) with the kanamycin resistance gene only, leaving the *nmb1869* promoter intact while P_*fHbp*_ was disrupted by changing the -10 sequence (TACCATAA to TACCATCC). Upstream and downstream fragments, and the kanamycin resistance cassette together with the modified -10 region mutation were amplified using the primer pairs prom-10-mut1-F/prom-10-mut1-R, prom-10-mut3-F/prom-10-mut3-R, prom-10-mut4-F/prom-10-mut4-R, prom-10-mut5-F/prom-10-mut5-R with genomic DNA as the target, while primers prom-10-mut2-F/prom-10-mut2-R, were used to amplify the kanamycin resistance cassette. PCR products were ligated into pGEM-T (Promega) following Gibson Assembly (NEB), and then digested with *Nco*I and *Not*I before being used to transform *N*. *meningitidis*.

Site-directed mutagenesis was performed with the Quickchange kit (Stratagene) according to the manufacturer’s protocol. To modify fHbp_9C_-GFP (introducing mut1, mut2, mut3, mut4, mut-αRBS-1, mut-αRBS-2 and mut-αRBS-1&2), oligonucleotides pairs mut1-F/mut1-R, mut2-F/mut2-R, mut3-F/mut3-R, mut4-F/mut4-R, mut-αRBS-1-F/mut-αRBS-1-R, mut-αRBS-2-F/Mut-αRBS-2-R, mut-αRBS-1&2-F/mut-αRBS-1&2-R were used respectively (Table 2). The reaction products were transformed into *E*. *coli* DH5α, and constructs were confirmed by sequencing.

Full length *fHbp* was amplified from *N*. *meningitidis* MC58 using primers fHbp(c)-F and fHbp(c)-R and ligated into pGEMT to yield p*fHbp*. Deletions were generated by PCR and the products were then ligated into pGEM-T (Promega) and transformed into *E*. *coli* DH5α. The identity of all constructs was confirmed by nucleotide sequencing.

Plasmids containing GFP fusions were generated by amplifying the promoter of *fHbp* with different lengths of the ORF with fHbp(TTS)-U or fHbp-GFP-F with either fHbp-GFP1C-R, fHbp-GFP5C-R or fHbp-GFP9C-R, and ligating the products into pEGFP-N_2_ (Clontech).

### RNA isolation and northern blot analysis

*N*. *meningitidis* was grown in liquid culture to mid. log phase, and RNA was isolated using the RNAeasy Miniprep Kit (Qiagen, UK) following the manufacturer’s protocol. For *E*. *coli*, bacteria were grown in liquid media to mid log phase, and RNA isolated using the FastRNA Pro Blue kit (MP Biomedicals) according to the manufacturer’s protocol. The purity and integrity of RNA were determined by gel electrophoresis and spectrophotometry.

For Northern blotting, 20 μg of total RNA was separated on a formaldehyde agarose gel prior to blotting as described previously [43], then transferred to Hybond N. Membranes were hybridised with ^32^P-ATP ɣ-labelled DNA fragments Northern blots were developed using a Fuji phosphorImager scanner. Probes for detecting *fHbp* and *tmRNA* were amplified from gDNA with primer pairs fHbp-U/fHbp-D, and tmRNA-U/tmRNA-D, respectively.

To analyse mRNA stability, *E*. *coli* containing relevant plasmids was grown in liquid culture to an OD_600nm_ of 0.5–0.6 then exposed to rifampicin (250 μg ml^-1^ final concentration) and incubated at 37°C with agitation. Bacteria were harvested for RNA isolation 0, 5, 10 and 20 minutes afterwards. Blots were probed with a labelled oligonucleotides GGTGCAGATGAACTTCAGGGTCAGCTTGCCGTAGGTGGCATCGCCCTCGC (to detect GFP mRNA), and a tmRNA product amplified from *E*. *coli* gDNA with primers Tm1 and Tm2 (Table 2). Band intensities of Northern blots were quantified using AIDA image analyzer software, standardised to the tmRNA loading control, and expressed as a ratio to the respective band intensities at t = 0 min.

### SDS-PAGE, western blotting and protein stability

Cell lysates were prepared by addition of SDS:PAGE loading buffer to an equal volume of bacteria obtained from liquid cultures by centrifugation. Total protein levels were measured using a Bradford assay and equal amounts of total protein loaded into each lane. Samples were boiled, then the proteins separated on polyacrylamide gels and transferred to immobolin P polyvinylidine fluoride (PVDF) membranes (Millipore, USA) using semi-dry transfer (Biorad, USA). For Western blot analysis, membranes were washed three times in 0.05% (w/v) dry milk/PBS with 0.05% (v/v) Tween-20 for 10 minutes, and then incubated with the primary antibody for one hour. Membranes were washed again three times and incubated for a further hour with a secondary, HRP-conjugated antibody. Binding was detected with an ECL Western Blotting Detection kit (Amersham, USA) and exposed to ECL Hyperfilm. An α-GFP mouse antibody (BD-living colors) was used at a final dilution of 1:8,000. α-fHbp polyclonal sera was used at a final dilution of 1:5000. α-RecA rabbit antibody (Abcam, UK) was used at a final dilution of 1:10,000. As secondary antibodies, goat α-rabbit or α -mouse IgG HRP-conjugated antibody (Dako, UK) was used at a final dilution of 1:10,000.

To determine protein stability, translation was prevented by adding spectinomycin (Sigma, final concentration 100 μg ml^-1^) to bacteria grown to an OD_600_  =  0.5 in liquid media. Samples were removed at times afterwards for Western blot analysis. Relative expression was calculated by measuring band intensities with ImageJ software, standardised to signals for loading controls (*i*.*e*. RecA or tmRNA), and shown as the ratio, relative to intensity of the control strain or condition.

### *In vitro* transcription/translation and prediction of RNA folding

For the template, DNA was isolated from derivatives of pEGFP-N_2_ containing one, five or nine codons of fHbp fused to GFP (GenElute Gel Extraction Kit, Sigma), linearised by digestion with *Not*I (New England Biolabs) and then purified using QIAquick (Qiagen). For RNA and DNA templates, 1 μg of nucleic acid was used as a template. The *in vitro* transcription/translation reaction was performed at 30°C, 37°C or 42°C for one hour using the *E*. *coli* S30 Extract system for Linear Templates *in vitro* Transcription/Translation Kit (Promega). The products were precipitated in acetone, then re-suspended in SDS-PAGE buffer prior to Western blot analysis.

RNA sequences were analysed using the *RNAfold* web server of the Vienna RNA package (http://rna.tbi.univie.ac.at/egi-bin/RNAfold.cgi). For each sequence, the minimum free energy in kcal mol^−1^ was predicted [44].

## Supporting Information

S1 Fig Thermoregulation of fHbp in *E*. *coli* occurs irrespective of the orientation in a plasmid. *E*. *coli* containing a plasmid with *fHbp* lacking the FNR box and P_*fhbp*_ but under the control of the SP6 promoter was grown at the temperatures indicated until mid-log, then protein was obtained from whole cell lysates and examined by Western blot using antibodies against fHbp and RecA (as a loading control).
(TIFF)

S2 Fig Stability of the GFP reporter and mRNA in *E*. *coli*. **(A)** Bacteria containing p*fHbp-1c-gfp* or p*fHbp-9c-gfp* was grown at 37°C to mid log phase, before protein synthesis was blocked by addition of spectinomycin. Samples were removed at indicated time-points and subjected Western blot analysis. Membranes were probed with antibodies recognising GFP or RecA (loading control). **(B)** Bacteria were harvested for RNA isolation after 5, 10 and 20 minutes of incubation at 37°C with agitation after the addition of rifampicin (250 μg/ml) to inhibit RNA synthesis. Total RNA (15 μg) from each sample were separated agarose formaldehyde gels and analysed by Northern blotting using probes for *gfp* and tmRNA. **(C)** Band intensities of the *gfp* transcripts were quantified using AIDA image analyzer software, standardised to the tmRNA loading control, and expressed as a ratio to the respective band intensities at t = 0 min.
(TIFF)

S3 Fig Putative RNA secondary structures of the *fHbp-nc-gfp* constructs. Colours of nucleotides show base-pair probabilities as calculated by the Vienna *RNAfold* programme (RBS indicated in blue and start codon in red).
(TIFF)

S4 Fig Modification of αRBS sequences does not affect mRNA stability. **(A)** After growth to mid-log phase, bacteria were harvested for RNA isolation after 5, 10 and 20 minutes of incubation at 37°C with agitation after the addition of rifampicin (250 μg/ml) to inhibit RNA synthesis. Total RNA (15 μg) from each sample were separated agarose formaldehyde gels and analysed by Northern blotting using probes for *gfp* and tmRNA. **(B)** Band intensities of the *gfp* transcripts were quantified using AIDA image analyzer software, standardised to the tmRNA loading control, and expressed as a ratio to the respective band intensities at t = 0 min.
(TIFF)

S5 Fig Modification of the a-RBSs in p*fHbp* influences thermosensing. **(A)** Mutagenesis of the mut-αRBSs in *fHbp*. Altered nucleotides are indicated in red, while the boxes show the predicted amino acid sequence. +1 indicates first nucleotide of the *fHbp* start codon. **(B)** Western blot analysis examining fHbp levels in *E*. *coli* containing modified pfHbp. Bacteria were grown at various temperatures overnight on solid media and protein isolated from whole cell lysates. Membranes were probed with antibodies recognising fHbp or RecA (loading control). **(C)** Quantification of fHbp levels performed by ImageJ analysis of Western blots probed with a-fHbp or a-RecA pAbs. Results normalised to the relative fHbp:RecA value obtained for pfHbp with mutαRBS-1 grown at 30°C as 100%. Experiments were performed on three indepdent occasions and error bars show the S.D.
(TIFF)

S6 Fig Modification of the a-RBSs in *N*. *meningitidis*. **(A)** Schematic diagram of the *fhbp* operon with the site of the insertion of the kanamycin resistance cassette. *fhbp* expression in *N*. *meningitidis* is regulated by two independent promoters (shown in arrows), yielding a 1,946 nt. bi-cistronic transcript and a 825 nt. mono-cistronic message generated from the promoter, P_*fHbp*_. The FNR box is in orange. Green dotted lines denote insertion site of an kanamycin cassette with its own terminator to disrupt the bi-cistronic transcript. **(B, C)** Western blot analysis examining fHbp levels in *N*. *meningitidis* Bacteria were grown at various temperatures overnight on solid media and protein isolated from whole cell lysates. Membranes were probed with antibodies recognising fHbp or RecA (loading control). Strains with fHbp under the control of the mono-cistronic (B) or bi-cistronic (C) *fHbp* promoters were examined. Construction of the strain lacking the monocistronic fHbp promoter is described in Fig 3A.
(TIFF)

S7 Fig Predicted secondary RNA structures for *modified fHbp* mRNAs. Colours of nucleotides show base-pair probabilities as calculated by the Vienna *RNAfold* programme, with the predicted minimum free energies in kcal/mole for *fHbp* mRNAs with a single C to G change in **(A)** αRBS1, **(B)** αRBS1, and **(C)** αRBS1 and αRBS2. The positions of the modified bases are shown.
(TIFF)
