# Supplementary figures and images for: Thermoregulation of Meningococcal fHbp, an Important Virulence Factor and Vaccine Antigen, Is Mediated by Anti-ribosomal Binding Site Sequences in the Open Reading Frame
Source: PLoS Pathog. 2016 Aug 25;12(8):e1005794. doi: 10.1371/journal.ppat.1005794 (PMC4999090; doi:10.1371/journal.ppat.1005794)

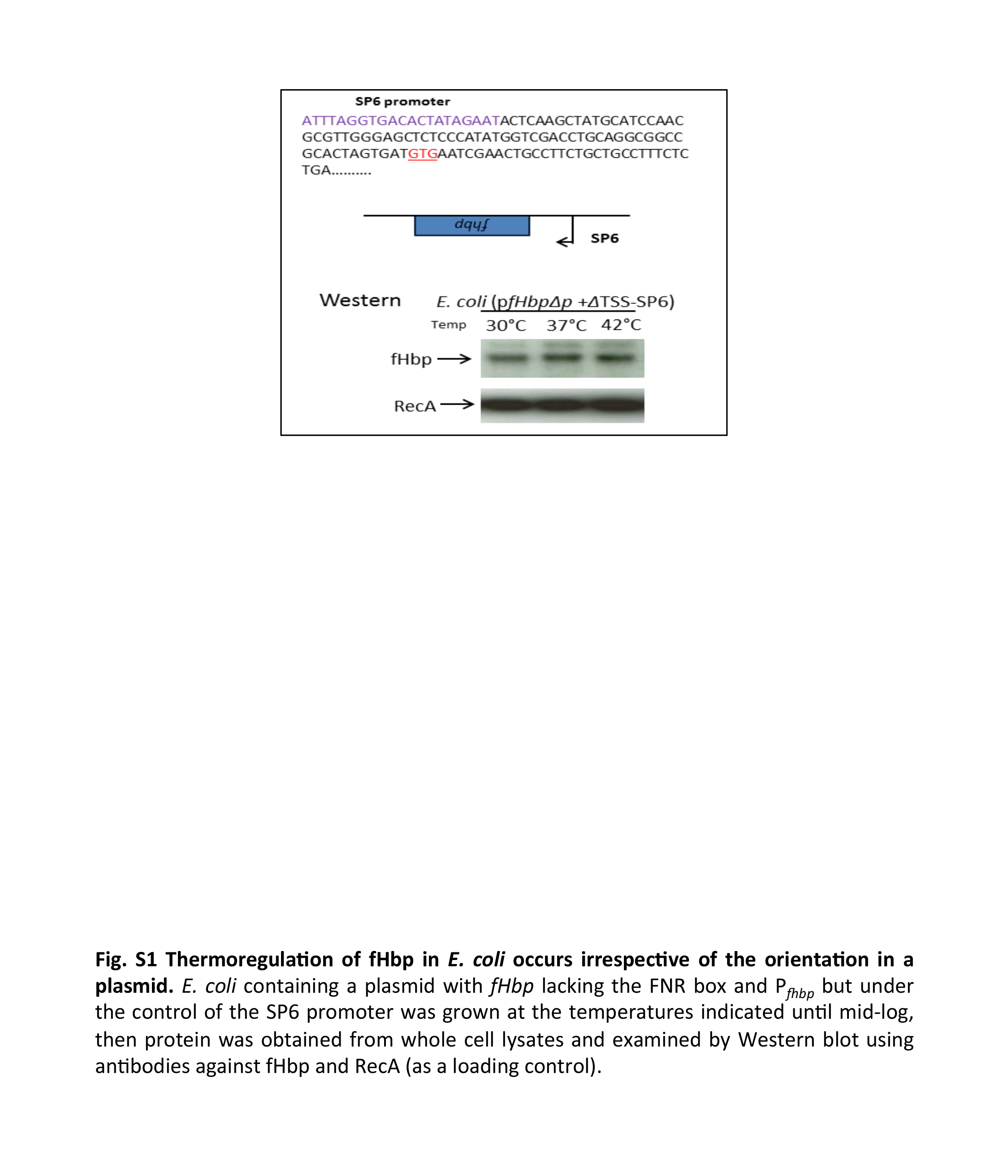

Supplement: S1 Fig — E. coli containing a plasmid with fHbp lacking the FNR box and Pfhbp but under the control of the SP6 promoter was grown at the temperatures indicated until mid-log, then protein was obtained from whole cell lysates and examined by Western blot using antibodies against fHbp and RecA (as a loading control). (TIFF) [file ppat.1005794.s001.tiff]

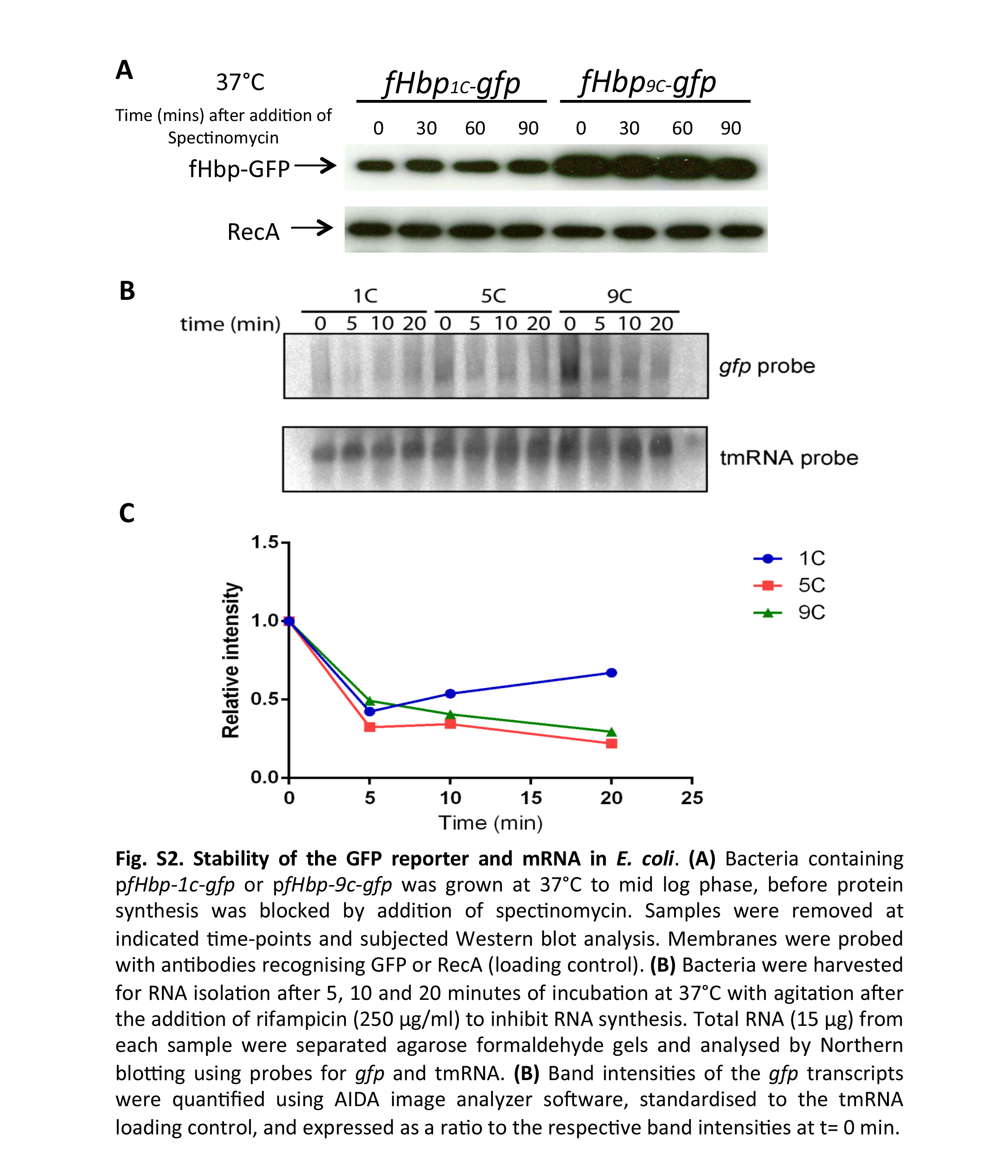

Supplement: S2 Fig — (A) Bacteria containing pfHbp-1c-gfp or pfHbp-9c-gfp was grown at 37°C to mid log phase, before protein synthesis was blocked by addition of spectinomycin. Samples were removed at indicated time-points and subjected Western blot analysis. Membranes were probed with antibodies recognising GFP or RecA (loading control). (B) Bacteria were harvested for RNA isolation after 5, 10 and 20 minutes of incubation at 37°C with agitation after the addition of rifampicin (250 μg/ml) to inhibit RNA synthesis. Total RNA (15 μg) from each sample were separated agarose formaldehyde gels and analysed by Northern blotting using probes for gfp and tmRNA. (C) Band intensities of the gfp transcripts were quantified using AIDA image analyzer software, standardised to the tmRNA loading control, and expressed as a ratio to the respective band intensities at t = 0 min. (TIFF) [file ppat.1005794.s002.tiff]

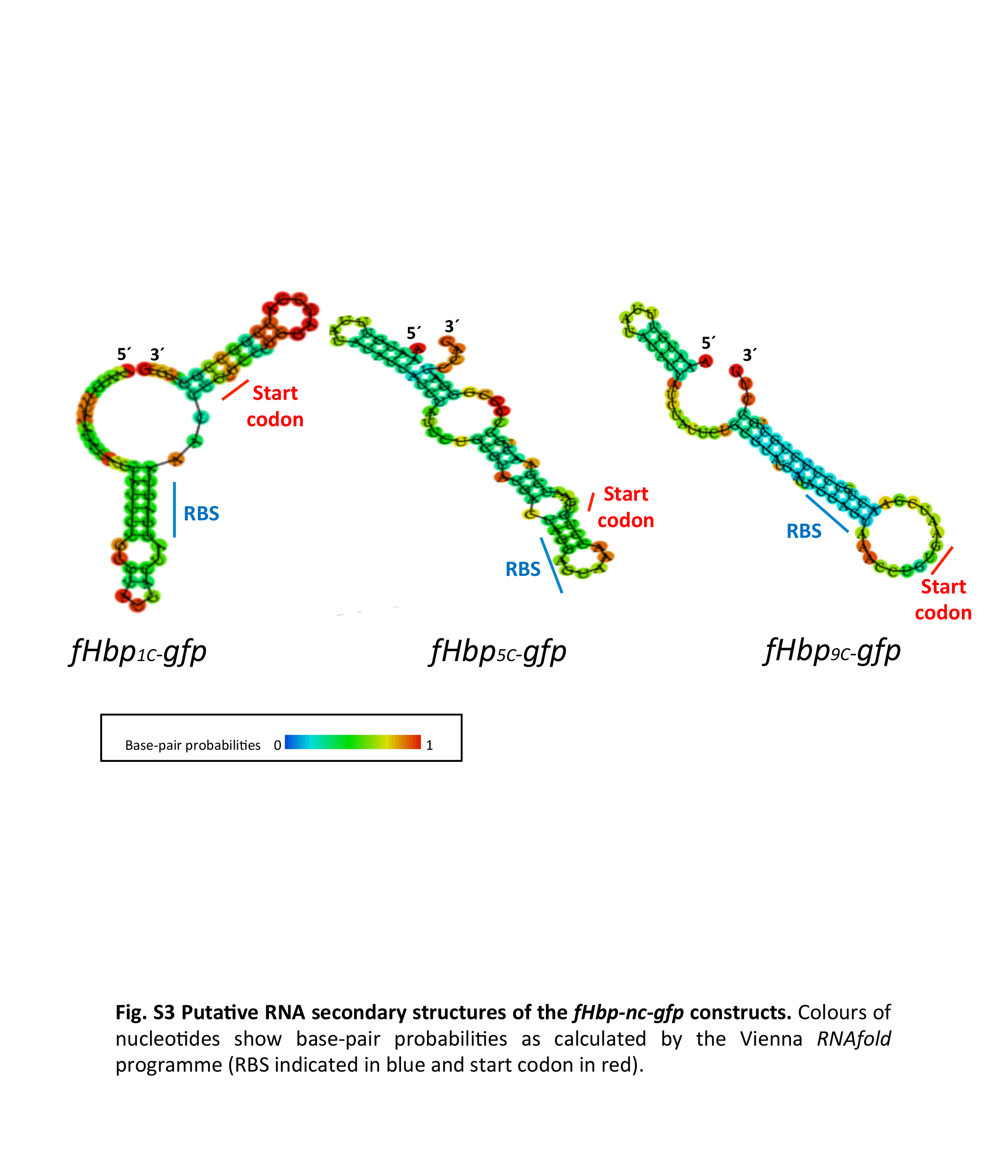

Supplement: S3 Fig — Colours of nucleotides show base-pair probabilities as calculated by the Vienna RNAfold programme (RBS indicated in blue and start codon in red). (TIFF) [file ppat.1005794.s003.tiff]

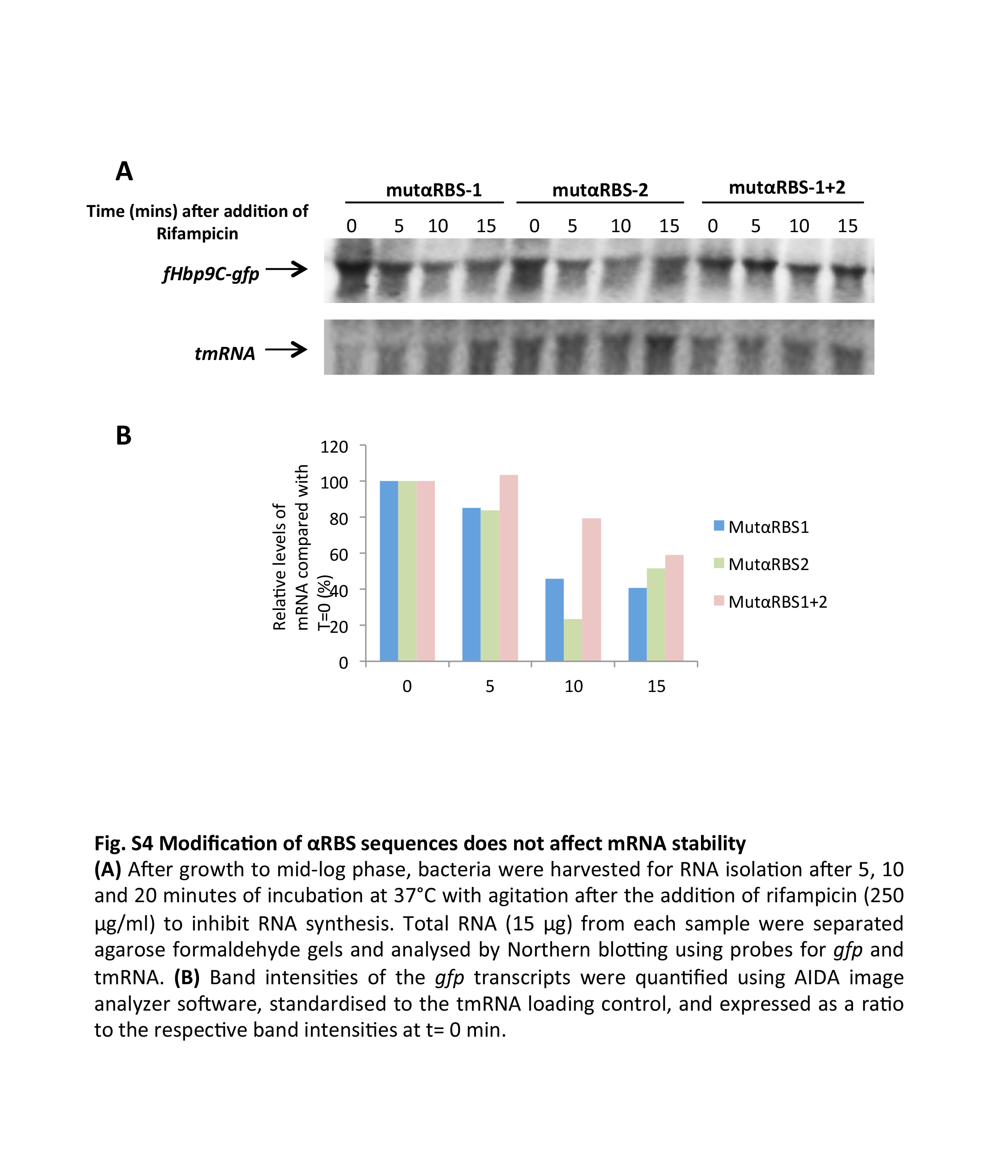

Supplement: S4 Fig — (A) After growth to mid-log phase, bacteria were harvested for RNA isolation after 5, 10 and 20 minutes of incubation at 37°C with agitation after the addition of rifampicin (250 μg/ml) to inhibit RNA synthesis. Total RNA (15 μg) from each sample were separated agarose formaldehyde gels and analysed by Northern blotting using probes for gfp and tmRNA. (B) Band intensities of the gfp transcripts were quantified using AIDA image analyzer software, standardised to the tmRNA loading control, and expressed as a ratio to the respective band intensities at t = 0 min. (TIFF) [file ppat.1005794.s004.tiff]

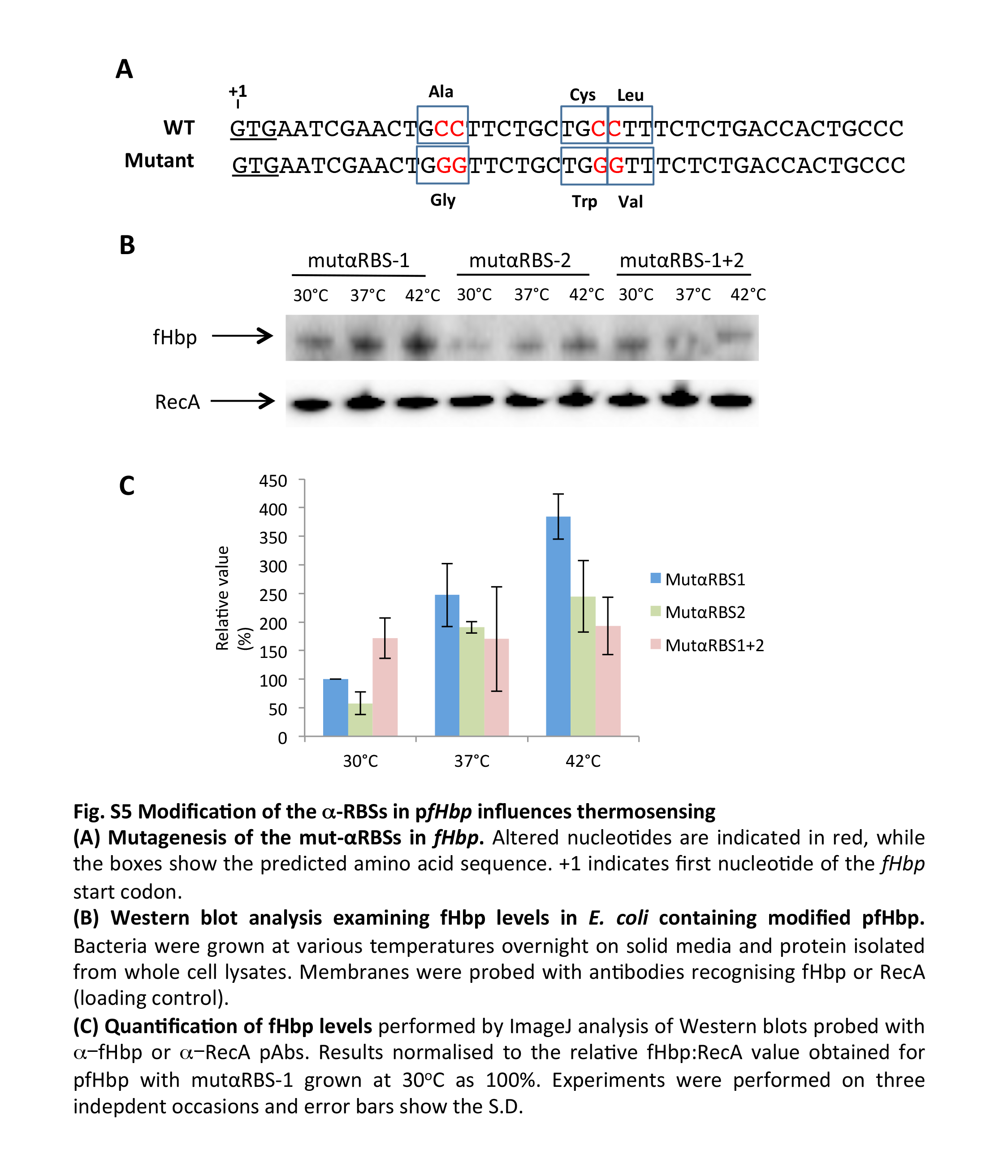

Supplement: S5 Fig — (A) Mutagenesis of the mut-αRBSs in fHbp. Altered nucleotides are indicated in red, while the boxes show the predicted amino acid sequence. +1 indicates first nucleotide of the fHbp start codon. (B) Western blot analysis examining fHbp levels in E. coli containing modified pfHbp. Bacteria were grown at various temperatures overnight on solid media and protein isolated from whole cell lysates. Membranes were probed with antibodies recognising fHbp or RecA (loading control). (C) Quantification of fHbp levels performed by ImageJ analysis of Western blots probed with a-fHbp or a-RecA pAbs. Results normalised to the relative fHbp:RecA value obtained for pfHbp with mutαRBS-1 grown at 30°C as 100%. Experiments were performed on three indepdent occasions and error bars show the S.D. (TIFF) [file ppat.1005794.s005.tiff]

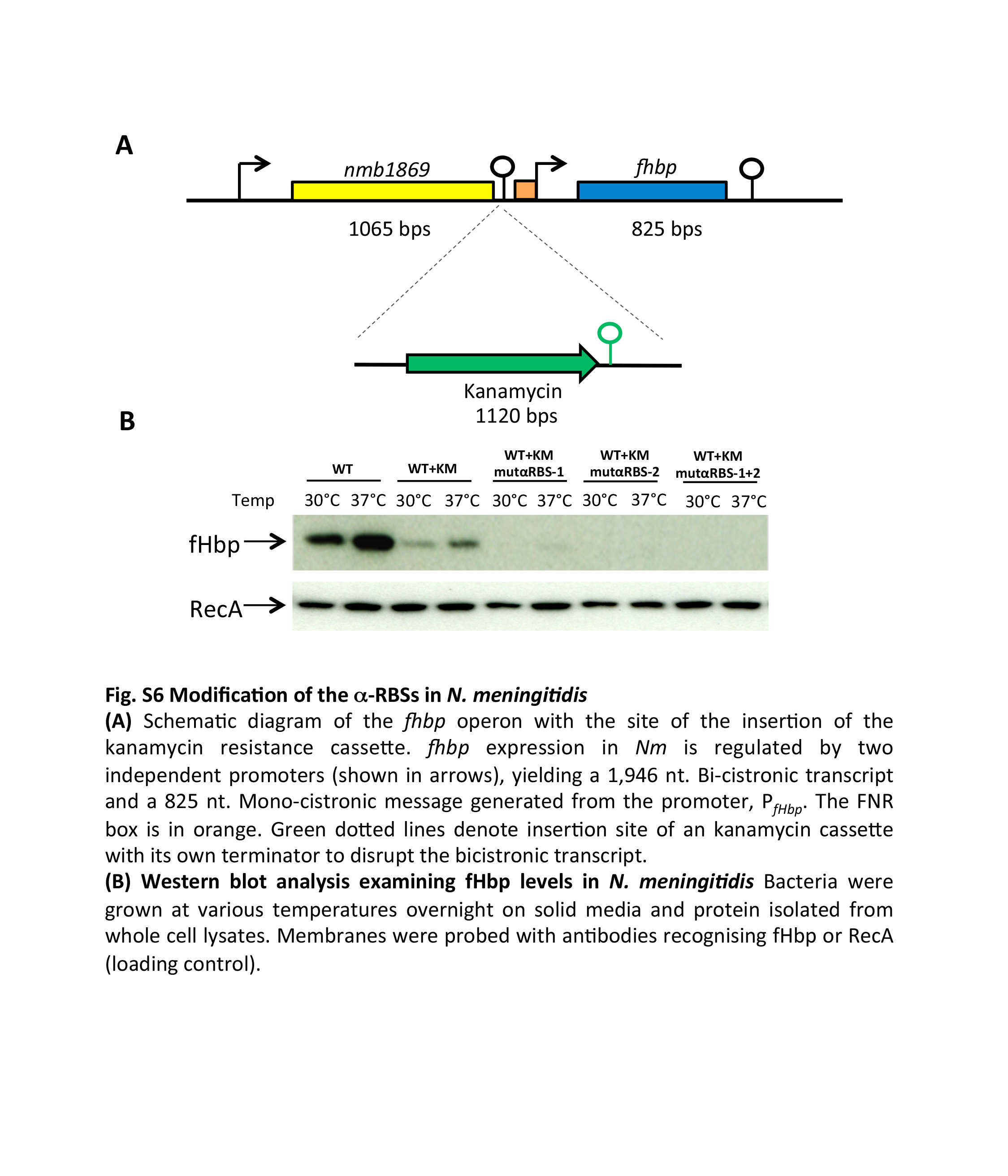

Supplement: S6 Fig — (A) Schematic diagram of the fhbp operon with the site of the insertion of the kanamycin resistance cassette. fhbp expression in N. meningitidis is regulated by two independent promoters (shown in arrows), yielding a 1,946 nt. bi-cistronic transcript and a 825 nt. mono-cistronic message generated from the promoter, PfHbp. The FNR box is in orange. Green dotted lines denote insertion site of an kanamycin cassette with its own terminator to disrupt the bi-cistronic transcript. (B, C) Western blot analysis examining fHbp levels in N. meningitidis Bacteria were grown at various temperatures overnight on solid media and protein isolated from whole cell lysates. Membranes were probed with antibodies recognising fHbp or RecA (loading control). Strains with fHbp under the control of the mono-cistronic (B) or bi-cistronic (C) fHbp promoters were examined. Construction of the strain lacking the monocistronic fHbp promoter is described in Fig 3A. (TIFF) [file ppat.1005794.s006.tiff]

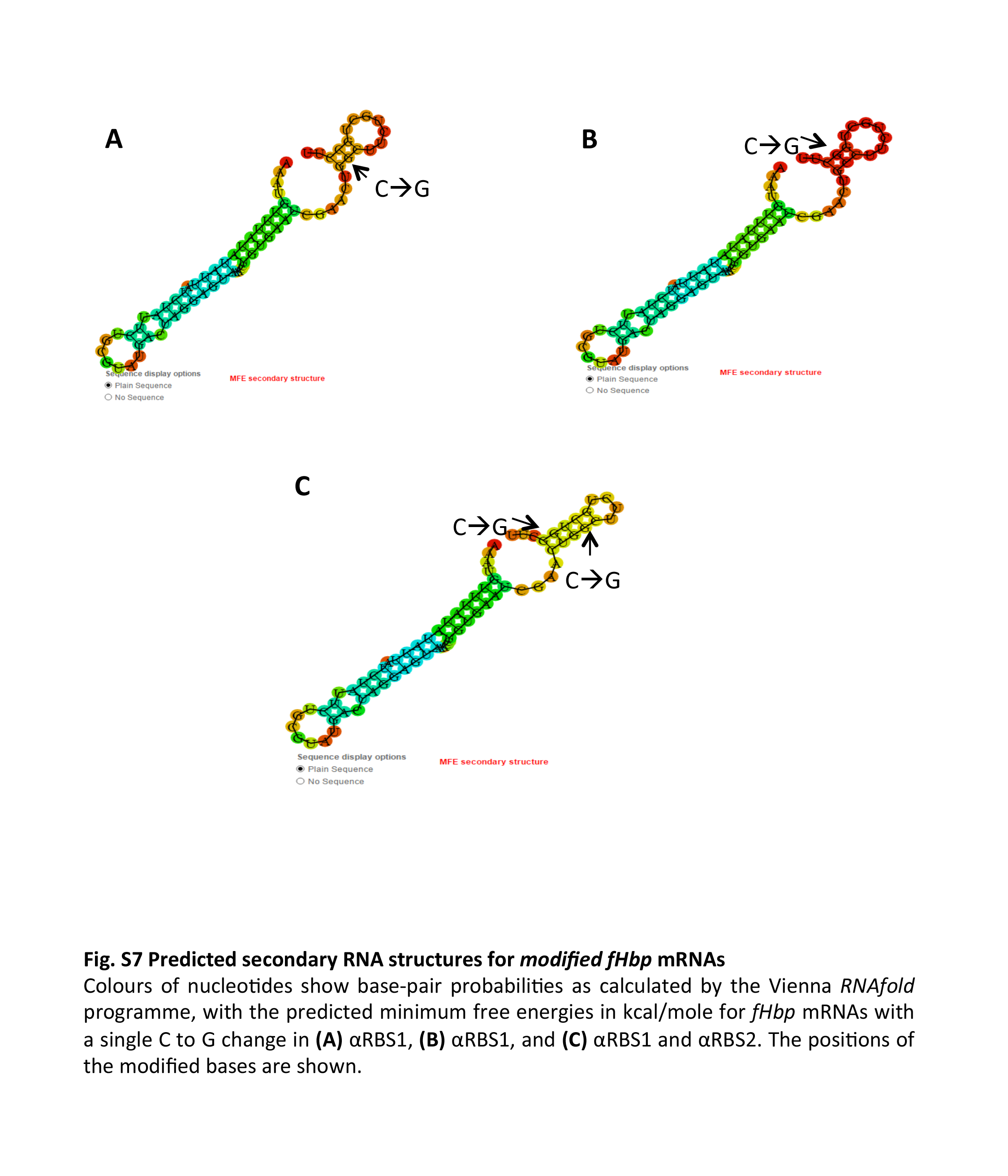

Supplement: S7 Fig — Colours of nucleotides show base-pair probabilities as calculated by the Vienna RNAfold programme, with the predicted minimum free energies in kcal/mole for fHbp mRNAs with a single C to G change in (A) αRBS1, (B) αRBS1, and (C) αRBS1 and αRBS2. The positions of the modified bases are shown. (TIFF) [file ppat.1005794.s007.tiff]
